# Analytical validation of a multi-cancer early detection test with cancer signal origin using a cell-free DNA–based targeted methylation assay

**DOI:** 10.1371/journal.pone.0283001

**Published:** 2023-04-14

**Authors:** Gregory E. Alexander, Wendy Lin, Fabian E. Ortega, Madhuvanthi Ramaiah, Byoungsok Jung, Lijuan Ji, Ekaterina Revenkova, Payal Shah, Christian Croisetiere, Jennifer R. Berman, Lane Eubank, Gunjan Naik, Jacqueline Brooks, Andrea Mich, Seyedmehdi Shojaee, Neda Ronaghi, Hemanshi Chawla, Xinyi Hou, Qinwen Liu, Christopher-James A. V. Yakym, Patriss Wais Moradi, Meredith Halks-Miller, Alexander M. Aravanis, Sonya Parpart-Li, Nathan Hunkapiller

**Affiliations:** GRAIL, LLC, A Subsidiary of Illumina, Inc., Currently Held Separate from Illumina, Inc., Under the Terms of the Interim Measures Order of the European Commission Dated 29 October 2021, Menlo Park, California, United States of America; Chinese University of Hong Kong, HONG KONG

## Abstract

The analytical validation is reported for a targeted methylation-based cell-free DNA multi-cancer early detection test designed to detect cancer and predict the cancer signal origin (tissue of origin). A machine-learning classifier was used to analyze the methylation patterns of >10^5^ genomic targets covering >1 million methylation sites. Analytical sensitivity (limit of detection [95% probability]) was characterized with respect to tumor content by expected variant allele frequency and was determined to be 0.07%-0.17% across five tumor cases and 0.51% for the lymphoid neoplasm case. Test specificity was 99.3% (95% confidence interval, 98.6–99.7%). In the reproducibility and repeatability study, results were consistent in 31/34 (91.2%) pairs with cancer and 17/17 (100%) pairs without cancer; between runs, results were concordant for 129/133 (97.0%) cancer and 37/37 (100%) non-cancer sample pairs. Across 3- to 100-ng input levels of cell-free DNA, cancer was detected in 157/182 (86.3%) cancer samples but not in any of the 62 non-cancer samples. In input titration tests, cancer signal origin was correctly predicted in all tumor samples detected as cancer. No cross-contamination events were observed. No potential interferent (hemoglobin, bilirubin, triglycerides, genomic DNA) affected performance. The results of this analytical validation study support continued clinical development of a targeted methylation cell-free DNA multi-cancer early detection test.

## Introduction

Currently, population-scale cancer screening is only recommended for a few cancers in the United States (US)—breast, cervical, colon, lung (in high-risk patients), and on a per-patient basis, prostate cancer [1–5], each of which is screened using a cancer-specific test (eg, mammography). Although these screening programs have helped to reduce the mortality of these cancers [6], unscreened cancers still account for about two-thirds of all cancer-related deaths in the US because many cases are diagnosed at late stages, when the probability of survival is lower [7]. In addition, the false positive rate of multiple single-cancer screening tests is cumulative [8], thus it is not feasible to screen for multiple cancers with a series of tests.

A blood-based multi-cancer early detection (MCED) test could address the unmet need for earlier detection of multiple cancers with a single, low, fixed false positive rate. Many cell-free DNA (cfDNA) cancer detection tests are being developed and a few are already in clinical use in the US and other countries [9, 10]. However, most of these tests only detect somatic tumor alterations that are specific to a single type of cancer, such as lung or colorectal cancer. Tumor-agnostic analyses of cfDNA (eg, whole-genome sequencing [WGS]) or approaches that combine cfDNA analysis with protein biomarkers may be more amenable for multi-cancer screening but lack accurate cancer signal origin (ie, tissue of origin) determination that could minimize subsequent diagnostic workup, may be confounded by cfDNA variants due to clonal hematopoiesis, and lack results from large clinical studies in intended-use populations [9, 11–14].

To address these challenges, a cfDNA MCED test is being studied in a clinical genomics program involving more than 145,000 participants in four prospective, multi-center clinical trials. Participants in the Circulating Cell-free Genome Atlas study (CCGA; NCT02889978) were divided into 3 approximately equal sized sub-cohorts designated by CCGA1, CCGA2 and CCGA3. Using the CCGA1 sub-cohort, it was demonstrated that whole-genome bisulfite sequencing outperformed WGS and targeted sequencing of short variants (eg, SNV [single nucleotide variants] and indels [insertion-deletion mutations]), and machine-learning classifiers to detect cancer and predict cancer signal origin were developed [15, 16]. Liu et al 2020 used the CCGA2 sub-cohort and showed that a targeted methylation-based MCED test simultaneously detected a cancer signal across >50 cancer types with a single low false positive rate of 0.7%, which is critical for population-level screening, and predicted cancer signal origin with 93% accuracy [17]. Further development and clinical validation of this test occurred using the CCGA3 sub-cohort [18] and are ongoing in three other studies (PATHFINDER [NCT04241796] interventional study designed to return results to healthcare providers and participants [19], STRIVE [NCT03085888], and SUMMIT [NCT03934866]). Here, the analytical validation, which is a series of non-clinical studies assessing the impact of sources of technical variation on the robustness or accuracy of assay results, is reported for the version of the MCED test that was used in Liu et al 2020 study and in the PATHFINDER interventional study.

## Materials and methods

### Ethics statement

The CCGA protocol and consent were reviewed and approved by the Institutional Review Board (IRB) or Independent Ethics Committee (IEC) for each of the 140 participating sites and the central IRB Western IRB (now WIRB Copernicus Group). The other IRBs/ethics boards were Hartford HealthCare IRB, Hartford, CT, US Oncology, Inc., IRB, The Woodlands, TX, Memorial Sloan Kettering Cancer Center Institutional Review Board/Privacy Board, New York, NY, University of Miami Institutional Review Board, Miami, FL, Mayo Clinic Institutional Review Board, Rochester, MN, Cleveland Clinic Foundation Institutional Review Board, Cleveland, OH, Avera Central Services IRB #3—Oncology IRB, Sioux Falls, SD, Lahey Clinic, Inc. Institutional Review Board, Burlington, MA, Biomedical Research Alliance of New York, Lake Success, NY, The Christ Hospital Institutional Review Board, Cincinnati, OH, University Health Network Research Ethics Board, Toronto, ON, Canada, Lehigh Valley Health Network’s Institutional Review Board, Allentown, PA, IntegReview Ethical Review Board, Austin, TX, and Dana-Farber Cancer Institute (DFCI) IRB, Boston, MA. IRBs provide oversight of the study throughout its duration. All participants provided written informed consent per regulatory requirements prior to participating in study-related activities and sample collection for the Circulating Cell-free Genome Atlas study (CCGA; NCT02889978), under which the analytical validation testing discussed herein falls.

### Test workflow

**Fig 1:**
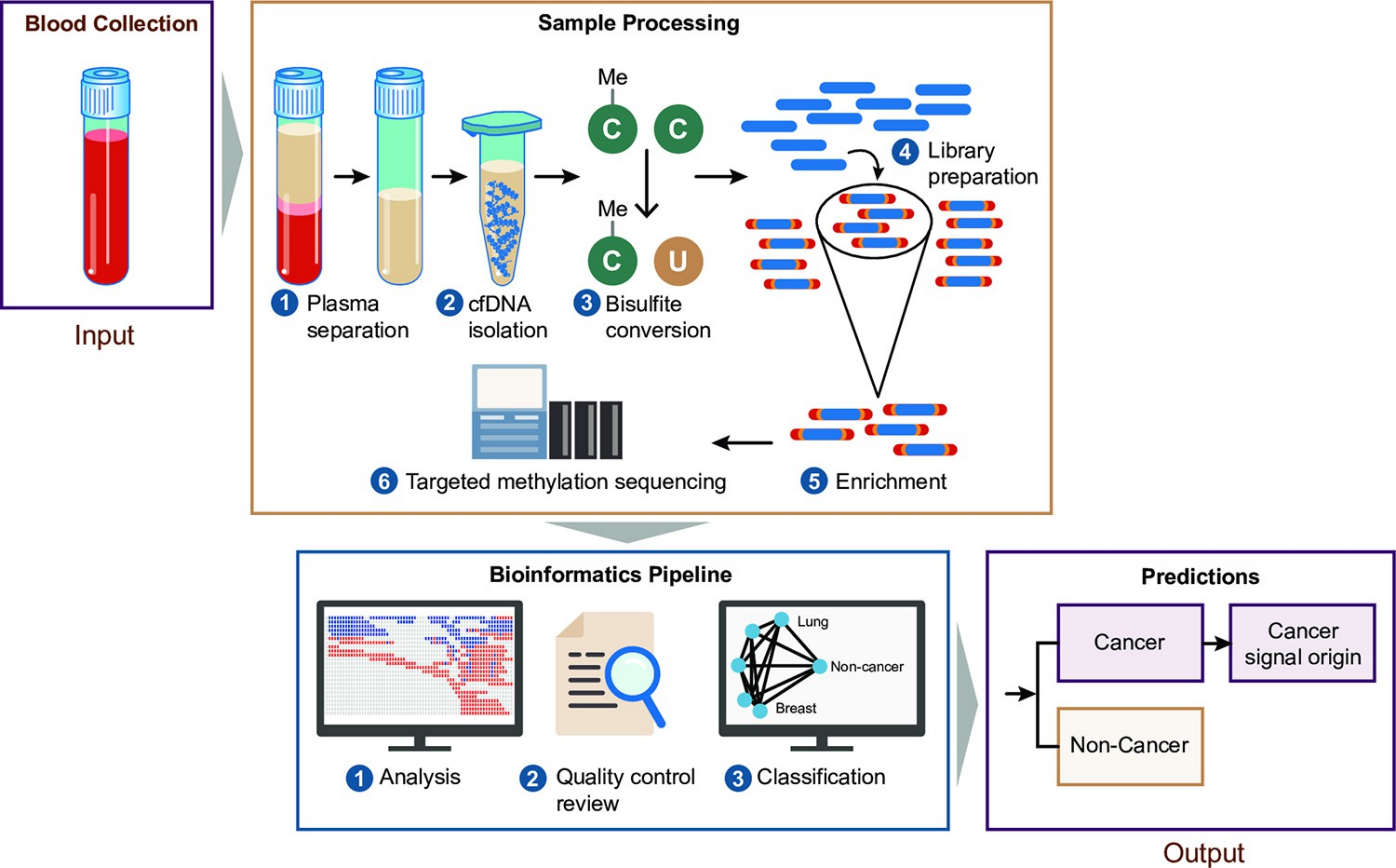
Overview of the multi-cancer early detection test workflow and computational pipeline. Cell-free DNA (cfDNA) fragments are isolated from plasma and are treated with bisulfite to distinguish methylation patterns. Sequencing libraries are generated, are enriched for >100,000 genomic regions previously identified as having cancer- and/or tissue-specific methylation patterns, and then undergo targeted methylation sequencing. Following an initial analysis, if the data pass a quality-control review, a machine-learning classifier analyzes the targeted methylation sequencing data from cfDNA fragments to determine cancer status and, if cancer is detected, predict the cancer signal origin (16). C, cytosine; cfDNA, circulating cell-free DNA; Me, methyl group; U, uracil.

The MCED test workflow (**Fig 1**) and computational pipeline have been described in detail [17]. Briefly, in the CCGA study, blood was collected into cfDNA blood collection tubes from participants who had not undergone any treatment before blood collection. Cell-free DNA was isolated from plasma using modified Automated MagMax kit (ThermoFisher Scientific, Waltham, MA; catalog #A29319). The median time from blood collection to plasma isolation was <2 days, and the median cfDNA yield was approximately 1.5 ng/mL blood from a 10 mL blood sample. Up to 75 ng cfDNA was bisulfite treated prior to generating dual-indexed sequencing libraries, which were enriched for >100,000 genomic regions previously identified as having cancer- and/or tissue-specific methylation patterns [15, 20]. The CpG sites within these genomic regions were targeted by hyper- and/or hypo-methylation probes. Enriched regions were sequenced using paired-end sequencing (Illumina Novaseq 6000, San Diego, CA; catalog #20012850). The median unique on-target coverage of the binary regions targeted by both hyper- and hypo-methylation probes was 139 reads per CpG. Coverage across binary target regions is an important metric of test performance.

Subsequently, in the bioinformatics pipeline, targeted methylation sequencing data were analyzed using source models of cancer-specific methylation patterns and an ensemble logistic regression classifier to generate a score. These models, along with thresholding parameters, were trained on samples divided into training and independent validation sets. Scores above the 99.4th percentile of those in non-cancer samples in the training set were considered cancer “signals” and predictive for cancer; those below this cutoff were predictive for non-cancer. Samples detected as cancer were further analyzed using source models of tissue-specific methylation patterns and a cancer signal origin classifier to predict the anatomic location of the primary tumor.

### Analytical validation

Five studies were performed to validate key elements in the MCED test workflow: test sensitivity and specificity, DNA input amount, reproducibility and repeatability, cross contamination, and potential interferents. In these studies, MCED test performance was assessed by the sensitivity and specificity of cancer detection and the accuracy of cancer signal origin prediction. Key metrics of cancer detection included binary classification score, binary target coverage, and abnormal coverage.

Binary target coverage is the coverage by unique cfDNA fragments across regions targeted by both hyper- and hypo-methylation probes (binary targets). The binary target regions were selected because they are minimally affected by the presence or absence of a strong cancer signal, and they therefore provide a stable measure of overall assay performance and sample quality applicable to both cancer and non-cancer samples. Binary target coverage was used for quality control purposes to verify that samples had sufficient genomic input.

**Fig 2:**
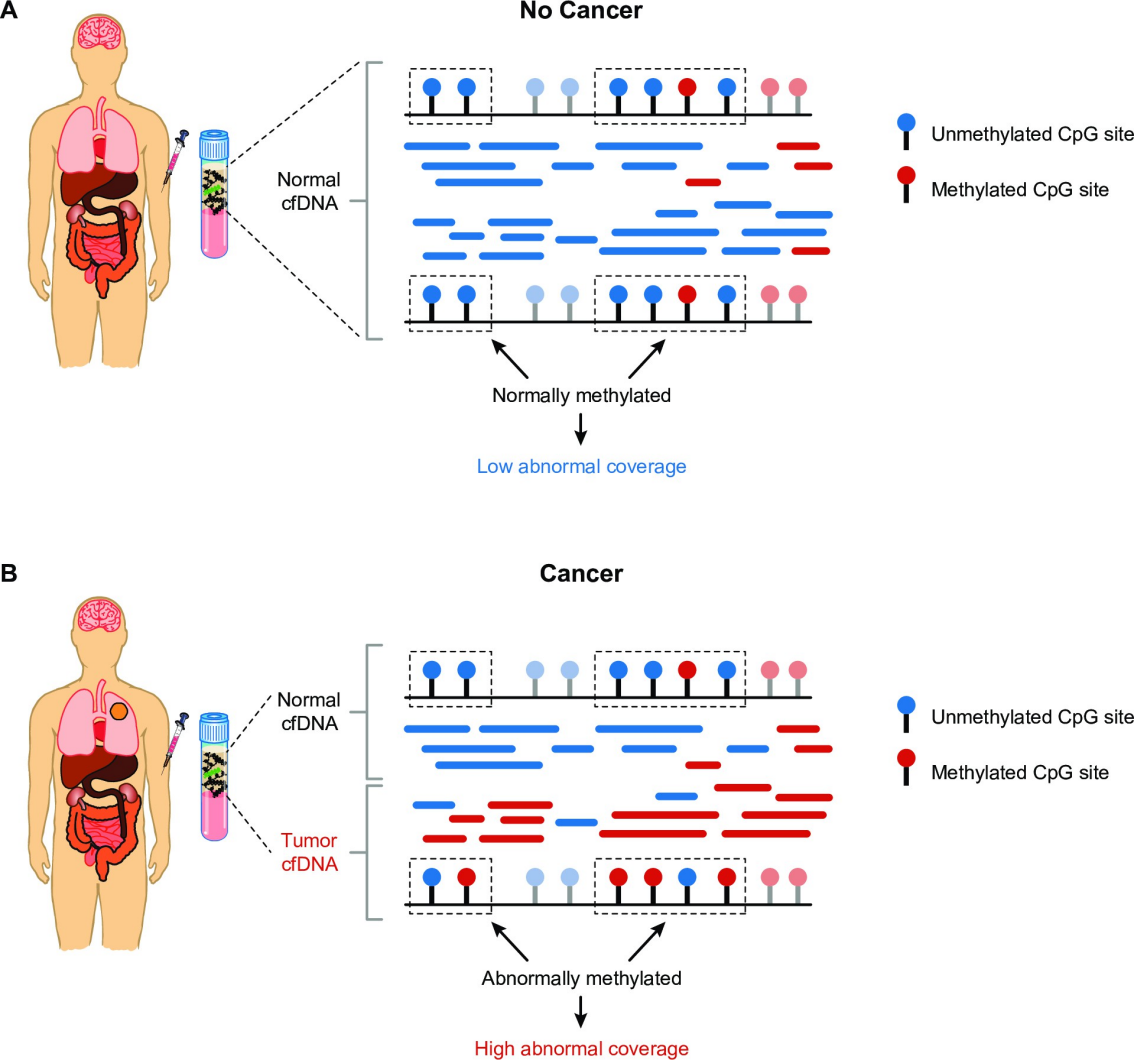
Abnormal coverage is a measure of abnormally methylated cell-free DNA (cfDNA) fragments analyzed by a targeted methylation-based multi-cancer early detection (MCED) test. In a healthy individual (A), plasma contains normal cfDNA shed by normal cells. In an individual with cancer (B; lung tumor represented by orange circle), plasma contains a mixture of normal and tumor cfDNA. Cell-free DNA fragments contain CpG methylation sites that may be either unmethylated (blue lollipops) or methylated (red lollipops), which are reflected in cfDNA sequencing reads (blue or red segments). In tumor cfDNA, some methylation regions are abnormally methylated (dotted rectangles in B), unlike cfDNA from individuals without cancer (A). The representation (coverage) of these abnormally methylated regions is quantified by abnormal coverage, which is a measure of cancer detection in the MCED test. The coverage of other regions that are not affected by cancer (light blue and light red lollipops) is quantified by binary target coverage, which is a measure of baseline test performance. cfDNA, circulating cell-free DNA.

Abnormal coverage is the coverage of unique cfDNA fragments likely to be abnormally methylated in cancer (**Fig 2**). The binary classification score is calibrated to be the percentile of a sample’s classifier-derived score among non-cancer samples in the training set. Cancer signal origin accuracy was defined as the proportion of known cancer samples with a correct signal origin prediction.

#### Analytical sensitivity and specificity

Cancer signal detection was determined by a machine-learning classifier analyzing methylation patterns across >100,000 genomic targets; therefore, it was not practical to employ a traditional definition of analytical limit of detection (LOD) as in the case of a test whose final output is detection of a single analyte or multiple single analytes. To address this challenge, expected variant allele frequency (VAF, defined as expected fraction of mutant reads in a cfDNA sample) was computed. A sample’s expected VAF was derived from processing samples on an independent DNA short variant detection assay and performing computations to quantify the level of tumor specific variant allele in the plasma relative to detected variants which were previously identified in matched tumor tissue for the same individual. A Bayesian model was applied to determine the frequency of mutated reads in a cfDNA sample across a set of somatic mutations identified in the participants’ tumor biopsy. Expected VAF is similarly computed elsewhere, described as circulating tumor allele fraction [21]. While short DNA variants are an orthogonal tumor-specific feature to the novel methylation features analyzed by the MCED test, this approach is justified because the available cancer signal in cfDNA should ultimately be driven by the fraction of cfDNA originating from the tumor. Analytical LOD was then established with respect to the expected VAF of test samples analyzed across serial dilutions and defined by the minimum required expected VAF for accurate classification in 95% percent of replicates. An additional advantage of this approach is that MCED test performance may be compared to tests that use a similar or different class of cancer specific features as long as the cancer signal available in cfDNA test samples is defined analogously in terms of expected VAF.

Analytical sensitivity was assessed from the cancer signal detection rate (proportion of samples called as cancer) across dilution series created by spiking cfDNA from cancer samples into cfDNA derived from individuals not known to have cancer (total 12.5 ng cfDNA). Six non-cancer samples were obtained from Discovery Life Sciences (Huntsville, AL; catalog #s 220130 and 220140) and 6 cancer samples were obtained from participants in the CCGA study with breast cancer (n = 1), colorectal cancer (n = 1), head and neck cancer (n = 1), lung cancer (n = 2), or lymphoid neoplasm (n = 1). For each cancer case, admixtures were prepared targeting 3 dilution levels around the lowest admixture fraction predicted from *in silico* titration to have 95% detection probability (LOD_95%_). Either 5 or 10 replicates of each targeted admixture level for each cancer case were then tested. All admixtures were processed using a total cfDNA input target of 12.5 ng, which represents the 30.5th percentile of the amount of extracted cfDNA observed in 15,893 samples that were in the training and holdout samples of CCGA.

Because DNA short variants were less frequent in the early-stage cancer cases in the CCGA cohort, which prevented the calculation of expected VAF and led to an underrepresentation of early-stage samples in our analysis of the LOD, additional evaluation of early-stage samples was performed. Both *in silico* titration and *in vitro* dilution analyses were performed, which were independent of small variants. For the *in silico* titration analysis, all detected stage I cancer samples from the training set of the CCGA2 sub-cohort were selected (n = 150) followed by a random sampling of 150 detected cancer samples from each of stages II, III and IV (some were independently processed samples from the same individual; **S1 Table**). Each *in silico* titration sample was simulated by mixing a random fraction of the cancer sample reads with non-cancer sample reads. The ratio of cancer sample reads to non-cancer sample reads is referred to as the *in silico* titration level, and the reciprocal ratio is referred to as the fold dilution. For each cancer sample, 3 *in silico* titration samples were generated for each *in silico* titration level. The *in silico* LOD was expressed as the lowest *in silico* titration level (corresponds to the highest fold dilution) that a source sample could undergo and retain 100% classification accuracy across 3 *in silico* titration sample replicates. The *in silico* LODs were binned according to fold dilutions falling within 1–1.33x, 1.33-2x, 2-4x, and >4x.

For the *in vitro* dilution analysis with early-stage cases, cfDNA samples from seven cancer participants of the CCGA2 sub-cohort (three stage I and four stage II) were selected based on the following criteria: stage I or II clinical diagnosis, consistent cancer detection from testing 2 independent plasma tubes, abnormal coverage ≤1 in order to be challenging, mean expected cfDNA yield of >25 ng from 2–3 plasma tubes and sufficient to create 80 replicates in total. The samples were diluted into pooled cfDNA extracted from individuals without cancer. Each cancer and non-cancer sample was processed in duplicate using 6ng of cfDNA input mass yielding a baseline result for cancer score and classification. Additionally, 2 to 20 replicates were processed for each diluted cancer plus non-cancer mixture sample yielding a total of 80 observations. The total input mass for diluted samples was 6ng with cfDNA from cancer samples ranging from 1.85 ng to 5 ng. A total of 72 plasma tubes from 43 unique non-cancer participants were selected from the CCGA2 sub-cohort with cfDNA yield >20 ng and abnormal coverage<0.05 or cfDNA yield >17 ng and abnormal coverage <0.08. All extracted non-cancer cfDNA was merged into one pool and used to dilute cancer cfDNA samples to generate the replicates (12 baseline non-cancer replicates were processed for the pool). Samples were run through the assay to characterize detection performance in early-stage cancer.

Specificity was calculated as 1 –false positive rate in 1204 samples (multiple tubes per participant) from a subset of CCGA samples from participants without cancer (n = 583), which were not used to train the classifier. Of note, all samples cannot be guaranteed to be true blanks (ie, devoid of cancer signals). As a result, the specificity calculation above was a conservative estimate of analytical specificity, which may be higher.

#### Input titration

To characterize test performance as a function of cfDNA input amount, cancer detection rates and cancer signal origin accuracy were assessed in six cancer samples (colorectal cancer, lung cancer, lymphoid neoplasm, multiple myeloma, upper gastrointestinal [GI] cancer, and renal cancer from CCGA study participants) and five commercially sourced non-cancer samples (StemExpress [Folsom, CA]; catalog # PBCUS080F, bioIVT [Hicksville, NY]; catalog # HUMANWBDBCT1805547). Non-cancer donors were selected with the following criteria: adult, non-smoking, HIV-, HBV-, HCV-negative. Seven different input amounts of cfDNA were tested (3, 5, 10, 20, 40, 75, and 100 ng) with 2–6 replicates at each level for cancer samples and 0–4 replicates at each level for non-cancer samples.

#### Reproducibility and repeatability

To determine whether test results were reproducible and repeatable, the concordance in the proportion of valid samples with correct cancer and cancer signal origin calls within and between runs was assessed across four runs performed by 15 operators using three groups of reagent lots and instruments as follows: with reagent lots designated as LOT 1, LOT 2, LOT 3 and instrument groups designated as Group_A, Group_B, Group_C, the four test runs were processed as Run1 = (Lot 1, Group_A), Run2 = (LOT 2, Group_B), Run3 = (Lot 3, Group_C), Run4 = (Lot 2, Group B). Different operators performed different processes within a run, each run lasting 6 to 7 days, with sample processing occurring over 4 weeks.

A total of 81 samples from 20 CCGA participants with cancer (breast [n = 8; stage II], anorectal/colorectal [n = 8; stages I, III], upper GI [n = 10; stages II, III], head and neck [n = 10; stage II], liver/bile duct [n = 6; stages II, III], lung [n = 8, stage III], lymphoid neoplasm [n = 8, stages II, III], multiple myeloma [n = 8, stage III], ovary [n = 8, stage III], and pancreas/gallbladder [n = 7, stage II]) and 45 samples from 15 randomly selected CCGA participants not known to have cancer and with 2–5 tubes of residual plasma were analyzed.

Samples were divided into two groups (high signal or low signal) based on a series of *in silico* titrations applied to sequence data previously generated in Liu et al 2020 [17]. Samples that remained detectable under high *in silico* dilution were defined as high signal, and those that did not were defined as low signal. For each type of cancer, a case from each group was included. Samples were selected from both the high signal and low signal categories to ensure that the cases included would encompass a range of cancer signal strength. Abnormal coverage was anticipated to be higher in the high-signal group than in either the low-signal or non-cancer groups.

Two replicates of all cancer samples and a subset of non-cancer samples were included in at least two independent runs to evaluate variability within-run (repeatability) and variability between runs (reproducibility).

#### Cross contamination

Three sets of experiments were performed to investigate the potential impact of cross contamination during sample handling and processing. First, to simulate potential cross contamination during plasma isolation, commercially sourced plasma samples from four individuals without cancer (bioIVT [Hicksville, NY] catalog # HUMANWBDBCT1805547; StemExpress [Folsom, CA] catalog # PBCUS080F) were mixed in pairs to determine the ability of the cross-contamination module [22] of the MCED test analysis software to detect the heterogeneity. This module applies a set of probabilistic models to allele frequencies observed across single-nucleotide polymorphisms (SNPs) to infer the presence of potentially superimposed genotypes and quantify the concordance of the SNP profiles and to determine the sex of the individual associated with the sample. Two pairs of plasma samples were tested; in each pair, one sample was designated as donor and the other as recipient. Donor plasma was spiked into recipient plasma at mass fractions near the expected LOD of the cross-contamination module (0.1%, 8 replicates; 0.2%, 14 replicates). Pure genome samples from each individual (2 replicates each) were also assessed as controls for negative contamination calls.

Second, pure genome (n = 24) and mixed genome (n = 150) samples from the sensitivity and specificity study were tested as negative and positive controls. Admixtures were generated by spiking cancer cfDNA samples (breast, colorectal, head and neck, lung, lymphoid neoplasm) into non-cancer cfDNA samples to simulate three expected VAF titration points for each cancer (0.2%, 0.6%, 1.2% for breast, colorectal, and lung case 1; 0.2%, 0.8%, and 1.2% for lung case 2; 0.1%, 0.6%, 1.2% for head and neck; 1%, 2%, 4% for lymphoid neoplasm).

Finally, the ability of the cross-contamination module to detect swapped samples and sex mismatch based on SNP genotype and sex chromosome concordances was evaluated by pairing samples from the input titration and reproducibility and repeatability studies. Pure genome samples were assessed as negative controls.

#### Potential interferents

To determine the impact of common endogenous substances that could potentially interfere with sample preparation or the performance of the MCED test, cfDNA recovery and test sensitivity and specificity were assessed in cancer admixtures spiked with hemoglobin, bilirubin, or triglycerides (MilliporeSigma, St. Louis, MO; hemoglobin: catalog # H7379; bilirubin: catalog # B4126; triglycerides: catalog # 17810). Because the MCED test analyzes short cfDNA fragments, high-molecular-weight genomic DNA was also tested as a potential interferent. Cancer admixtures were generated by spiking plasma samples from individuals not known to have cancer (n = 50, StemExpress [Folsom, CA]; catalog # PBCUS080F, bioIVT [Hicksville, NY]; catalog # HUMANWBDBCT1805547) with abnormally methylated DNA from human HCT116 DKO cells (Zymo Research, Irvine, CA; catalog # D5014-1 and D5014-2) at 1% of total cfDNA extracted from unspiked samples. Each interferent was spiked into non-cancer samples and cancer admixtures at 5 different levels (hemoglobin: 0, 100, 500, 1000, 2000 mg/dL; bilirubin: 0, 5, 10, 15, 20 mg/dL; triglycerides: 0, 100, 200, 400, 500 mg/dL; genomic DNA: 0, 50, 100, 150, 200% of total cfDNA extracted from unspiked samples) with 5 replicates at each level.

Cell-free DNA recovery was quantified using AccuClear DNA quantitation assay (Biotium, Fremont, CA; catalog # 31029). Test sensitivity and specificity were assessed by the false negative rate (proportion of cancer admixtures not detected as cancer) and false positive rate (proportion of non-cancer samples detected as cancer), respectively.

## Results

### Participant disposition

The disposition of the 39 individual cancer participants from the CCGA study are summarized in **Table 1**. Altogether, participants encompassed a total of 12 cancer signal origins (head and neck, lung, lymphoid neoplasm, breast, colorectal, multiple myeloma, upper GI, renal, pancreas/gallbladder, anorectal, liver/bile duct, and ovary).

**Table 1:** Participant disposition by analytical validation study.

Study	Sample/Participant ID	Cancer Type	Clinical Stage	Histological Group	Duration Between Diagnosis and Blood Draw, Days	Race	Age	Sex	Smoking Status
**LOD**	5	Lung	III	NSCLC squamous	35	White, non-Hispanic	61	Male	Former smoker
**LOD**	2	Lung	III	NSCLC adenocarcinoma	22	White, non-Hispanic	70	Male	Former smoker
**LOD**	1	Head and neck	IV	Squamous cell carcinoma	32	Hispanic	63	Male	Former smoker
**LOD**	3	Lymphoid neoplasm	IV	Mature B-cell neoplasms	7	White, non-Hispanic	72	Female	Non-smoker
**LOD**	4	Breast	IV	Other/missing	14	White, non-Hispanic	57	Female	Current smoker
**LOD**	6	Colorectal	IV	Adenocarcinoma	6	White, non-Hispanic	57	Female	Non-smoker
**LOD-S**	7	Colorectal	I	Adenocarcinoma	13	White, non-Hispanic	55	Female	Former smoker
**LOD-S**	8	Pancreas gallbladder	II	Carcinoma	14	White, non-Hispanic	50	Male	Current smoker
**LOD-S**	9	Head and neck	II	Squamous cell carcinoma	45	Hispanic	44	Male	Non-smoker
**LOD-S**	10	Upper GI	II	Adenocarcinoma	24	White, non-Hispanic	50	Male	Current smoker
**LOD-S**	11	Lung	II	NSCLC Carcinoma	8	White, non-Hispanic	70	Male	Former smoker
**LOD-S**	12	Lung	I	SCLS	22	Black, non-Hispanic	67	Female	Former smoker
**LOD-S**	13	Head and neck	I	Squamous cell carcinoma	40	Hispanic	61	Male	Current smoker
**IT**	14	Multiple myeloma	Non-informative	Plasma cell tumor	0	White, non-Hispanic	71	Male	Former smoker
**IT**	15	Lymphoid neoplasm	Non-informative	B lymphoblastic	0	White, non-Hispanic	33	Male	Former smoker
**IT**	16	Lung	III	Malignant neoplasm	6	White, non-Hispanic	72	Female	Former smoker
**IT**	17	Colorectal	IV	Adenocarcinoma	8	White, non-Hispanic	53	Female	Former smoker
**IT**	18	Upper GI	IV	Neuroendocrine	22	White, non-Hispanic	58	Male	Non-smoker
**IT**	19	Renal	IV	Renal cell carcinoma	-5	White, non-Hispanic	58	Female	Former smoker
**RR**	32	Anorectal	I	Squamous cell carcinoma	27	White, non-Hispanic	64	Female	Former smoker
**RR**	20	Head and neck	II	Squamous cell carcinoma	20	White, non-Hispanic	56	Male	Former smoker
**RR**	21	Upper GI	II	Adenocarcinoma	32	White, non-Hispanic	65	Female	Former smoker
**RR**	26	Lymphoid neoplasm	II	Mature B-cell neoplasms	50	White, non-Hispanic	74	Male	Former smoker
**RR**	28	Pancreas gallbladder	II	Adenocarcinoma	12	White, non-Hispanic	69	Female	Non-smoker
**RR**	29	Pancreas gallbladder	II	Adenocarcinoma	13	White, non-Hispanic	69	Male	Non-smoker
**RR**	30	Head and neck	II	Squamous cell carcinoma	37	White, non-Hispanic	65	Male	Former smoker
**RR**	33	Breast	II	Adenocarcinoma	40	White, non-Hispanic	42	Female	Former smoker
**RR**	44	Breast	II	Adenocarcinoma	8	White, non-Hispanic	55	Female	Non-smoker
**RR**	50	Liver bile duct	II	Hepatocellular carcinoma	10	Black, non-Hispanic	63	Male	Former smoker
**RR**	22	Lymphoid neoplasm	III	Mature B-cell neoplasms	0	White, non-Hispanic	73	Male	Former smoker
**RR**	24	Lung	III	SCLC	7	White, non-Hispanic	78	Female	Former smoker
**RR**	25	Upper GI	III	Adenocarcinoma	19	White, non-Hispanic	69	Female	Non-smoker
**RR**	27	Lung	III	SCLC	69	White, non-Hispanic	65	Female	Current smoker
**RR**	31	Colorectal	III	Adenocarcinoma	7	White, non-Hispanic	56	Male	Current smoker
**RR**	34	Liver bile duct	III	Hepatocellular carcinoma	57	White, non-Hispanic	61	Male	Non-smoker
**RR**	36	Multiple myeloma	III	Plasma cell tumor	15	White, non-Hispanic	74	Male	Former smoker
**RR**	39	Multiple myeloma	III	Plasma cell tumor	16	White, non-Hispanic	52	Male	Non-smoker
**RR**	46	Ovary	III	Adenocarcinoma	15	White, non-Hispanic	57	Female	Other/ missing
**RR**	49	Ovary	III	Adenocarcinoma, carcinoma	22	Black, non-Hispanic	73	Female	Non-smoker

GI, gastrointestinal; IT, input titration; LOD, level of detection; LOD-S, supplementary LOD analysis using additional early-stage cancer cases; NSCLC, non-small cell lung cancer; RR, reproducibility and repeatability; SCLC, small-cell lung carcinoma.

### Analytical validation

#### Analytical sensitivity and specificity

**Fig 3:**
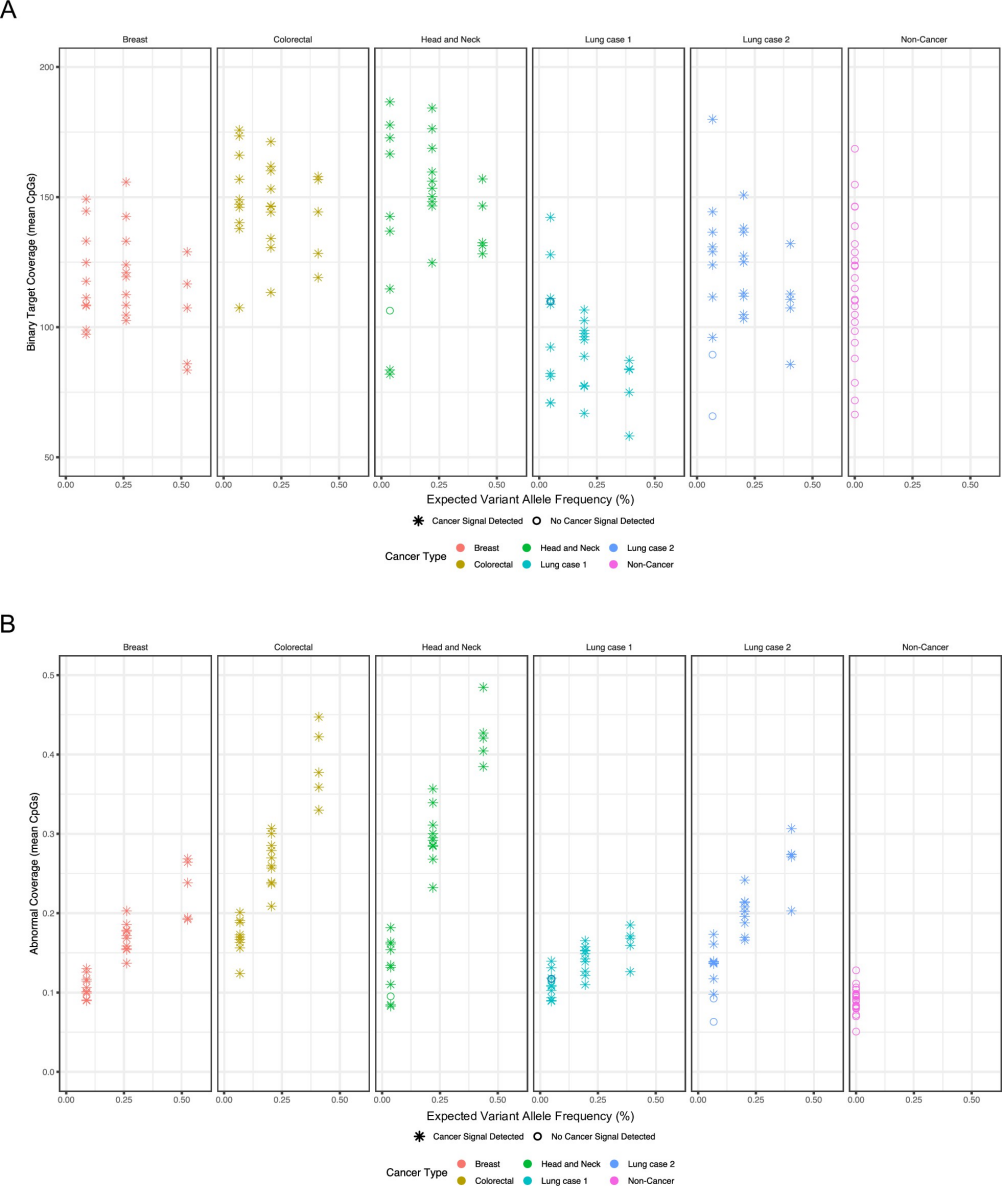
Binary target coverage is not correlated with variant allele frequency while abnormal coverage is correlated with variant allele frequency. (A) Binary target coverage, which quantifies the coverage of methylation regions in cell-free DNA (cfDNA) fragments that are not affected by cancer, is a measure of the baseline performance of the multi-cancer early detection test. Binary target coverage is not affected by tumor content of admixtures (proportion of cfDNA fragments with variants identified in samples with matched tumor biopsy samples) in breast cancer (red), colorectal cancer (olive), head and neck cancer (green), and lung cancer (teal and blue) samples, similar to non-cancer samples (magenta). Samples with a cancer signal detected or not detected are indicated by asterisks or open circles, respectively. (B) Abnormal coverage is correlated with variant allele frequency (proportion of cfDNA fragments with variants identified in samples with matched tumor biopsy samples) in breast cancer (red), colorectal cancer (olive), head and neck cancer (green), and lung cancer (teal and blue) samples. Samples called as cancer or non-cancer are indicated by asterisks or open circles, respectively.

As expected, binary classification scores were positively correlated with expected VAF (**S1 Fig**), but binary target coverage was not (**Fig 3A**). Similar to binary classification scores, abnormal coverage increased as expected VAF increased (**Fig 3B**).

For cancer detection with correct cancer signal origin prediction, LOD_95%_ with respect to expected VAF estimated by linear interpolation when analyzed separately by case ranged from 0.07% to 0.17% across five tumor cases and was 0.51% for the lymphoid neoplasm case (**Table 2**). The data are summarized by detection rate observed for each level of expected VAF tested (hit rates) in **S2 Table**.

**Table 2:** LOD_95%_ with respect to expected VAF (%) for 5 cancers.

Cancer	Cancer Detection, LOD_95%_ Expected VAF (%)	Cancer Detection + Cancer Signal Origin Prediction, LOD_95%_ Expected VAF (%)
**Breast**	≤0.09	≤0.09
**Colorectal**	≤0.07	≤0.07
**Head/Neck**	0.13	0.13
**Lung Case 1**	0.12	0.16
**Lung Case 2**	0.17	0.17
**Lymphoid Neoplasm**	0.51	0.51

LOD_95%_, limit of detection (95% probability) with respect to expected variant allele frequency (VAF). Computed using linear interpolation. There were 10 replicates tested at each level bounding the interpolated LOD.

Six non-cancer samples tested in quadruplicate were correctly classified as non-cancer in 24/24 tests (100% specificity; 95% confidence interval [CI], 85.8–100%). In addition, among 1204 non-cancer samples from 583 unique individuals without cancer, 1195 were correctly classified as non-cancer (99.3% specificity; 95% CI, 98.6–99.7%) (**S3 Table**). Among the 9 false positives, 8 were from the same 4 participants testing positive twice. For these 4 participants, the cancer signal origin prediction was the same for both replicates from each sample. For the 1 participant who had 1 sample identified as cancer and 1 sample identified as non-cancer, the top cancer signal origin was the same for both samples, suggesting that the underlying signal is an attribute of the sample and not due to an aberrant processing issue.

**Fig 4:**
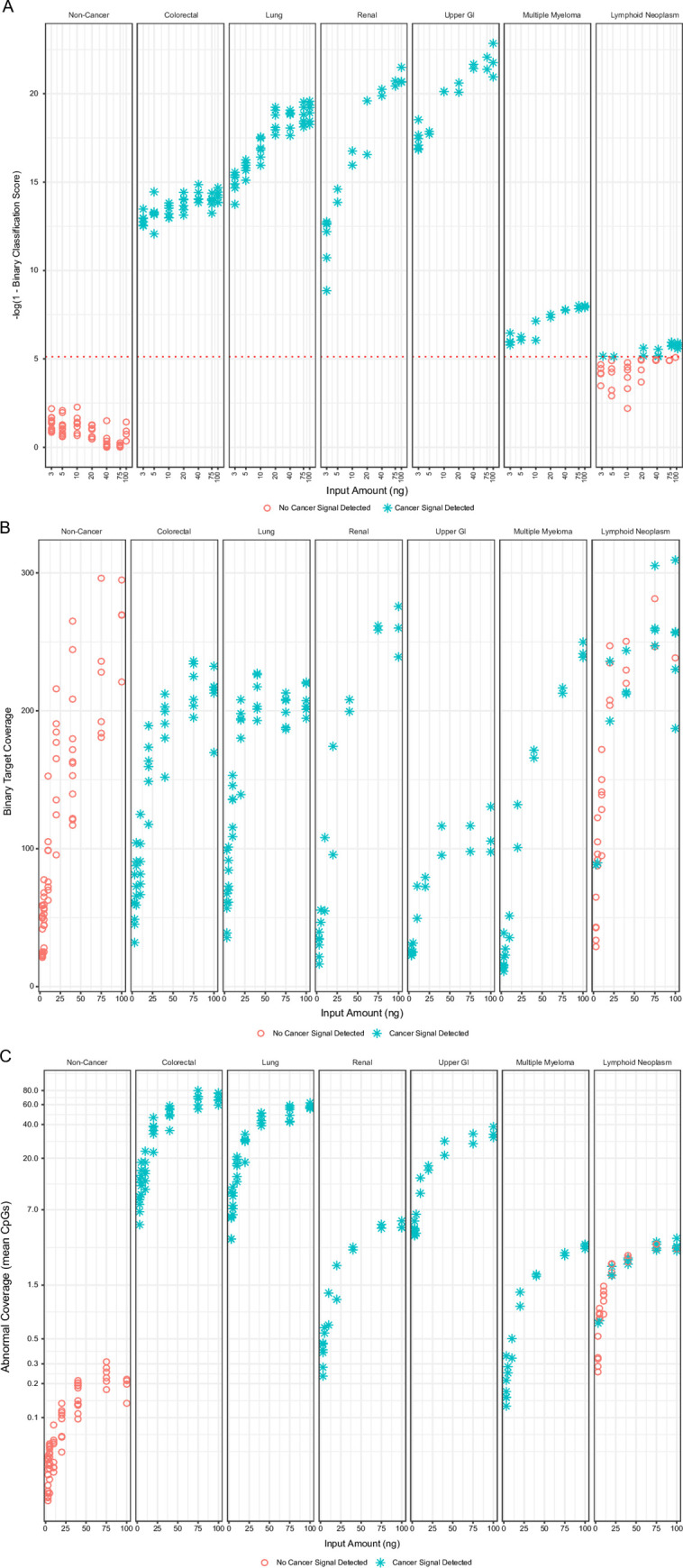
Correlations between cfDNA input amount, cancer type, and (A) binary classification score, (B) binary target coverage, and (C) abnormal coverage. Samples called as cancer or non-cancer are indicated by asterisks or open circles, respectively. (A) Binary classification score is positively correlated with the cell-free DNA input amount in the multi-cancer early detection test in colorectal, lung, renal, and upper gastrointestinal cancer and multiple myeloma. Scores for non-cancer samples are low and well below the detection cut-off (red dotted line). (B) Binary target coverage is positively correlated with the cell-free DNA input amount in the multi-cancer early detection test for both cancer (colorectal, lung, renal, and upper gastrointestinal [GI] cancer and multiple myeloma) and non-cancer samples. (C) Abnormal coverage is positively correlated (nonlinearly) with cfDNA input amount and generally lower in non-cancer samples than cancer samples. GI, gastrointestinal.

The cancer types and clinical stages for the 600 samples evaluated in the *in silico* titration analysis are shown in **S1 Table**. Results of the analysis by stage are shown in **S2 Fig**, which depicts the frequency of detected samples by stage at each fold dilution. Most samples could be highly diluted and still remain detectable as cancer as demonstrated by the bars in the two highest-fold dilution categories on the right-hand side of the figure (2-4x dilution and >4x dilution). A total of 55%, 70%, 76%, and 90%, respectively, from stage I, II, III, and IV cancers could withstand a 2-fold or greater dilution and remain detectable. Results of the in vitro dilution analysis of 7 additional early-stage cancer cases are shown in **Table 3** and **S3 Fig**. Six of seven of these cases showed 100% detection across all replicates despite a 2-fold or greater dilution; one stage I lung cancer, which was diluted 3.2-fold, had 53% detection across 19 replicates (**Table 3**). **S3 Fig** shows cancer scores resulting for all observations of cancer samples either diluted or undiluted. Generally, even with dilution and a 2-3-fold lower than typical DNA input level, resulting cancer scores consistently fell above the cancer signal detection threshold. In one case (stage I lung), the dilution resulted in wide variation in scores across replicates.

**Table 3:** *In vitro* dilution analysis of additional early-stage cases.

Sample/Participant ID	Cancer Type	Clinical Stage	Cancer sample cfDNA/total cfDNA (ng)	Fold Dilution	Number of Replicates	Percentage of Replicates Detected (%)
**7**	Colorectal	I	3/6	2.0	12	100
**12**	Lung	I	1.85/6	3.2	19	53
**13**	Head and neck	I	3/6	2.0	8	100
**9**	Head and neck	II	5/6	1.2	8	100
**8**	Pancreas gallbladder	II	1.6/6	3.8	20	100
**11**	Lung	II	3.8/6	1.6	10	100
**10**	Upper GI	II	5/6	1.2	2	100

#### Input titration

Across tumor types, binary classification score and binary target coverage were positively correlated with input cfDNA amount (**Fig 4A and 4B**). Abnormal coverage increased with cfDNA input amount across all samples up to approximately 75 ng cfDNA; as expected, the abnormal coverage was generally lower in non-cancer samples than cancer samples (**Fig 4C**).

Of the 245 samples tested, one 75 ng sample (0.4%) was excluded from analysis because it was called as contaminated. Across all input levels, cancer was not detected in any non-cancer samples (n = 62). Cancer was detected in all replicates across all input levels (3 ng to 100 ng) in 5/6 cancers (colorectal, 42/42; lung, 42/42; multiple myeloma, 19/19; renal, 19/19; upper GI, 19/19). For the lymphoid neoplasm, cancer was detected in 16/41 replicates. As lymphoid neoplasm cfDNA input increased from 3 to 100 ng, detection rates increased from 16.7% to 83.3%.

Overall test sensitivity was 86.3% (157/182) for 6 cancers and specificity was 100% (62/62) for five non-cancer samples. Cancer signal origin was correctly predicted in all samples with cancer signal detected (157/157).

#### Reproducibility and repeatability

For overall sample concordance with clinical diagnosis, a total of 126 samples (81 cancer, 45 non-cancer) were tested. Across four test runs with three groups of reagent lots and instruments, cancer was called correctly in 77/81 (95.1% concordant with diagnosis) cancer samples and 45/45 (100% concordant with diagnosis) non-cancer samples. For samples with cancer signal detected, cancer signal origin prediction was correct in 76/77 (98.7% concordant with diagnosis).

In within-run tests, cancer was called consistently in 31/34 (91.2%) sample pairs. For non-cancer samples, cancer status was consistently predicted as non-cancer in 17/17 (100%) sample pairs. In cases where cancer was detected, the cancer signal origin was called consistently in 30/31 (96.8%) sample pairs. Among between-run tests, pairwise concordance for cancer signal detection was 129/133 (97.0%) and 37/37 (100%) for cancer and non-cancer sample pairs, respectively. Cancer signal origin predictions were concordant in 128/129 (99.2%) of samples with cancer signal detected (n = 129).

**Fig 5:**
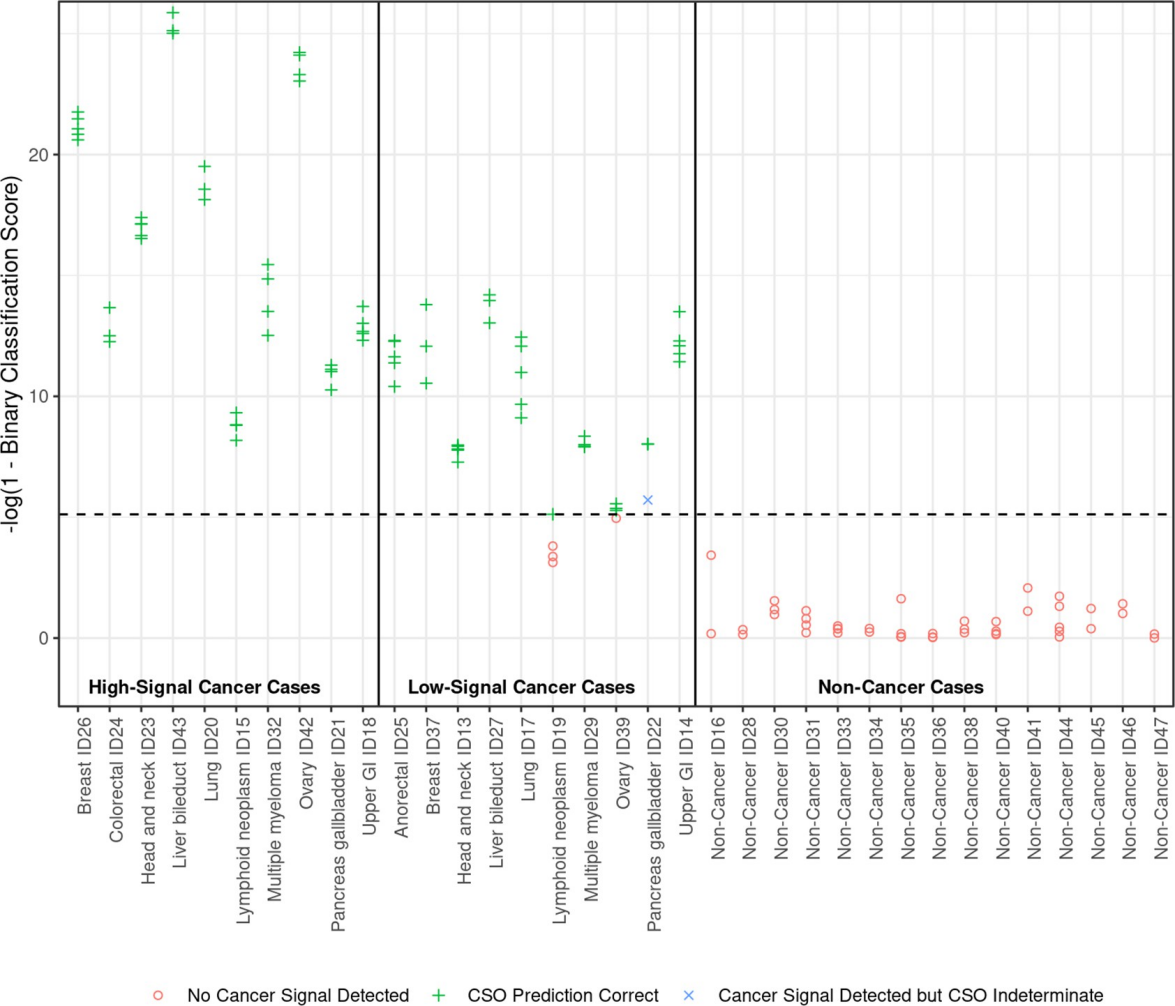
Binary classification scores of cancer and non-cancer samples were visually clustered in 3 groups. The binary classification score of a sample is the percentile of its classifier-derived score among non-cancer samples in the training set. Binary classification scores were log transformed as shown on the y-axis to facilitate visualization. The horizontal dotted line indicates the threshold score used to call samples as cancer (indicated with a “+” if tissue of origin was predicted correctly or “×” if it was predicted as indeterminate) or non-cancer (red open circles). GI, gastrointestinal; ID, sample identifier.

Consistent with these findings, binary classification scores (**Fig 5**) across replicates in cancer and non-cancer samples tended to cluster in groups defined by participants. Non-cancer samples (**Fig 5**, *right panel*) tended to have lower binary classification scores than cancer samples (*left* and *center panels*).

#### Cross contamination

In the plasma mixture analysis, the cross-contamination module of the MCED test called as contaminated 8/14 (57.1%) non-cancer recipient plasma samples spiked with non-cancer donor plasma samples at 0.2% by mass and 0/8 samples spiked at 0.1%. None (0/8) of the pure genome samples were identified as contaminated.

In the cfDNA mixture analysis, 150/150 non-cancer samples spiked with cancer samples were flagged as contaminated at all expected VAF titration points, demonstrating the contamination detection module has an LOD_95%_ of at least 0.2% by mass ratio. None (0/24) of the pure genome unspiked samples were identified as contaminated.

**Fig 6:**
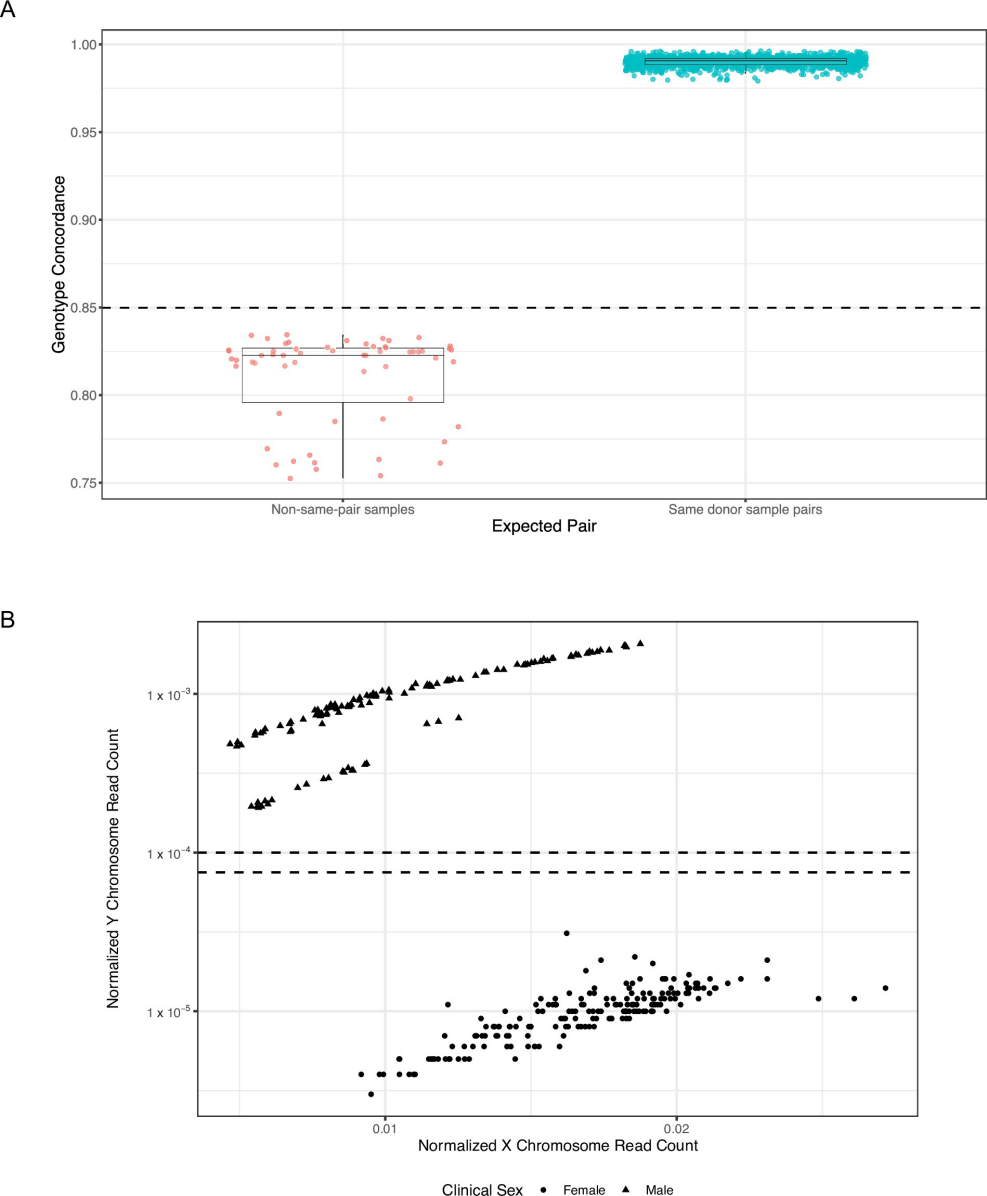
Assessment of cross contamination in plasma samples with the cross-contamination module of the bioinformatics pipeline. (A) Concordance of single-nucleotide polymorphism genotypes in same-donor sample pairs (teal circles) and non–same-pair samples (red circles). The horizontal dotted line indicates the threshold of 85% concordance used to call samples as not concordant (swapped) or concordant (not swapped). Boxes indicate 25th and 75th percentiles and the line inside corresponds to the median. Whiskers extend to minimum and maximum values, excluding outliers. Data points have been slightly offset horizontally (jittered) to better visualize points that may otherwise overlap. (B) Sex calls analysis. Male (triangles) and female (circles) samples were differentiated by the proportion of X and Y chromosome sequencing reads (x- and y-axes, respectively). The horizontal dotted lines indicate the thresholds used to call samples as male or female.

Sample swap analyses demonstrated 100% genotype concordance for 1380 replicates from the same donor (“same-pair” samples) and 60 replicates from different donors (“non–same-pair” samples). Moreover, all genotype concordances for same-donor sample pairs exceeded the concordance threshold of 0.85, whereas those for non–same-pair samples were below this threshold (**Fig 6A**).

In the sex call analysis, 189/189 female samples were called as female and 119/119 male samples were called as male (**Fig 6B**).

#### Potential interferents

**Fig 7:**
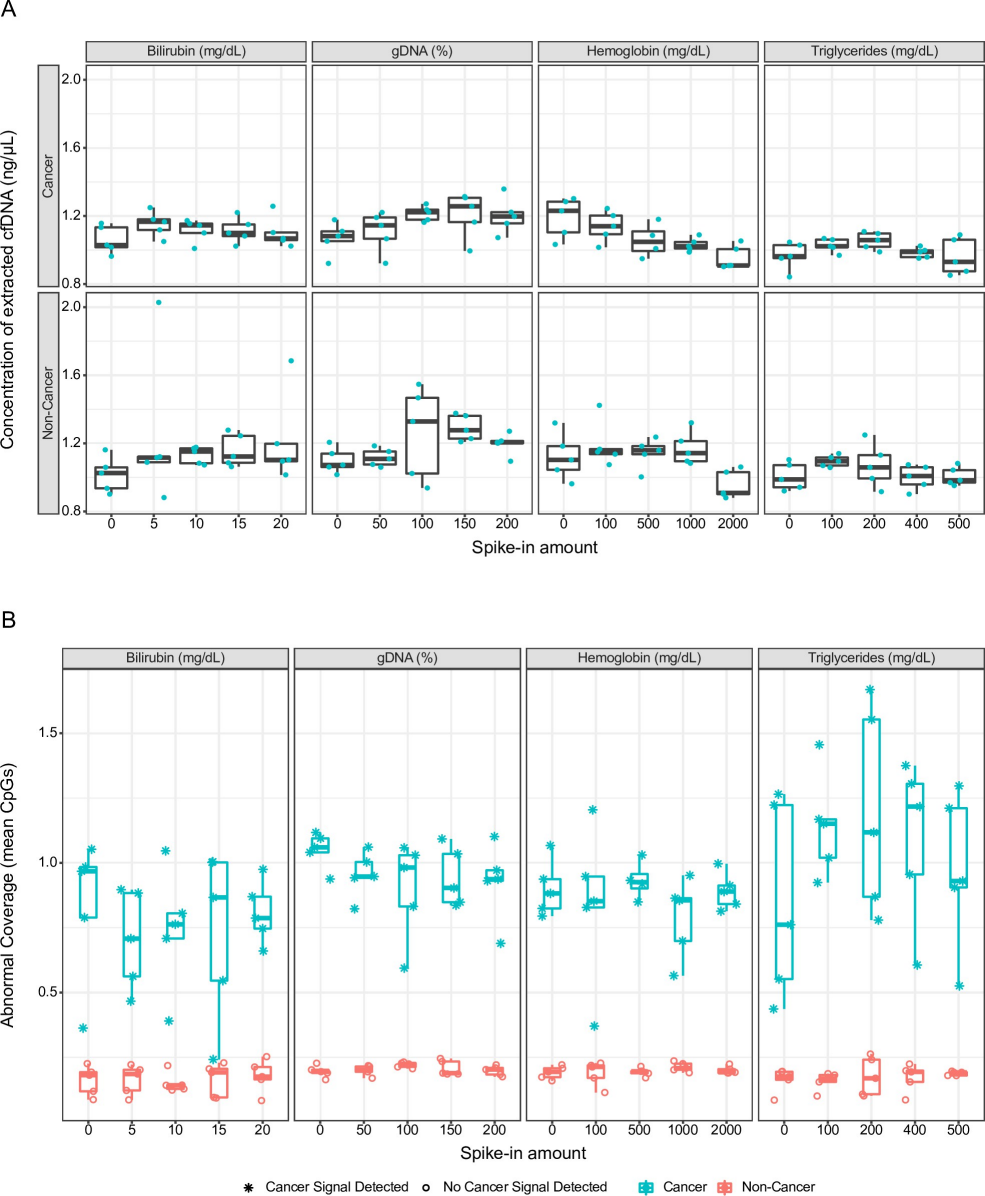
Effect of various concentrations of 4 potential interferents on (A) concentration of extracted cell-free DNA (cfDNA) and (B) abnormal coverage. Prior to cfDNA extraction, non-cancer plasma samples and cancer admixtures were spiked with bilirubin (0–20 mg/dL), high-molecular-weight genomic DNA (0–200% of total cfDNA extracted from unspiked samples), hemoglobin (0–2000 mg/dL), and triglycerides (0–500 mg/dL). Cancer admixtures were generated by adding abnormally methylated DNA from human HCT116 DKO cells to non-cancer plasma samples. Teal circles in Panel A represent samples. Boxes indicate 25th and 75th percentiles and the line inside corresponds to the median. Whiskers extend to minimum and maximum values, excluding outliers. gDNA, genomic DNA. Samples called as cancer or non-cancer are indicated by asterisks or open circles, respectively. Data points have been slightly offset horizontally (jittered) to better visualize points that may otherwise overlap.

The impact of 4 potential interferents on cfDNA isolation was assessed. The average concentration of DNA extracted from cancer admixtures tended to decrease as spiked-in hemoglobin concentration increased (**Fig 7A**). Without spiked-in hemoglobin, average DNA concentration was 1.2 ng/μL but only 0.95 ng/μL in the presence of 2000 mg/dL hemoglobin. However, spiked-in hemoglobin did not affect non-cancer samples. The presence of bilirubin, triglycerides, and genomic DNA in plasma samples did not affect cfDNA recovery (**Fig 7A**).

None of the tested interferents affected abnormal coverage (**Fig 7B**), binary target coverage (**S4 Fig**), or test sensitivity or specificity. A cancer signal was detected for all cancer admixtures (99/99) and not for any of the non-cancer samples (0/100).

## Discussion

Analytic validation consists of a series of non-clinical studies focused on assessing the impact of sources of technical variation on the robustness or accuracy of assay results. Collectively, the test samples used for analytical validation are not intended to be representative of clinical covariates of a particular intended use population but rather to only be representative of general expected specimen characteristics. The performance of a targeted methylation cfDNA-based MCED test was evaluated in 5 analytical validation studies with source specimens derived from 39 cancer participants that encompass a total of 12 distinct cancer signal origins (head and neck, lung, lymphoid neoplasm, breast, colorectal, multiple myeloma, upper GI, renal, pancreas/gallbladder, anorectal, liver/bile duct, and ovary). An additional 600 cancer samples (all stages) from 460 individuals were assessed using an *in silico* titration analysis.

In these studies, the MCED test achieved high specificity (99.3%) and accurately predicted cancer signal origin with high reproducibility and repeatability. In addition, the test workflow was robust across diverse conditions. Test performance was consistent for 3 to 75 ng of input cfDNA in all five non-hematological cancers. The hematological cancer was a borderline case with regard to cancer signal detection; false negatives occurred across the input range and therefore were not driven by input alone. Test performance varied somewhat by stage, and there was, as expected, more often a strong signal from later-stage cancers. Test performance was not affected by four potential interferents in blood, and cross-contamination and sample swaps were correctly detected in more than 1400 sample pairs.

Analytical validation of multivariate assays, such as this MCED test, is challenging because the evaluation of test sensitivity using a single analyte-based limit of detection experiment is not directly applicable [23] due to the fact that the MCED test aggregates information from >100,000 genomic regions to assess cancer signal status. As an alternative, test sensitivity with respect to expected VAF of tumor mutations in a sample has been characterized. A sample’s expected VAF was derived from processing samples with an independent DNA short variant detection assay and performing computations to quantify the level of tumor specific variant allele in the plasma. These tumor specific variant alleles were previously identified using matched tumor tissue from the same individual. Although the MCED test analyzes cancer signatures derived from methylation markers and not DNA short variants, it is assumed that plasma samples with high expected VAF (high tumor allele content and consequently high tumor derived cfDNA content) will be associated with higher methylation-derived classification scores. The LOD experimental design in this manuscript characterizes analytical detection sensitivity in terms of the lowest expected VAF across specimen replicates that resulted in positive cancer signal detection and correct cancer signal origin predictions for at least 95% of replicates. The LOD is not intended as a measure of the clinical sensitivity to be expected in a given population since the clinical performance of an assay will be affected by many clinical factors such as frequency at which ctDNA is shed in detectable quantities into the bloodstream of individuals in the given population of interest.

DNA short variants were less frequent in the early-stage cancer cases in the CCGA cohort, which prevented the calculation of expected VAF and led to an underrepresentation of early-stage samples in our analysis of the LOD. Consequently, evaluation of early-stage samples using in silico titration and in vitro dilution analyses, which are independent of DNA short variants, was performed. Overall, the *in silico* analysis indicates that many samples from early-stage cancers (stage I and stage II) are highly dilutable without loss of cancer signal detection or CSO prediction accuracy. These findings were substantiated by results from *in vitro* testing performed at dilution levels indicated by the *in silico* analysis. For the *in vitro* dilution analysis, samples were processed with a 1.2-fold to 3.8-fold dilution while using as little as 1.85 ng to 5 ng of cancer cfDNA input per reaction, which is about three-fold less than the typical input available with from single tube of blood. Under these challenging processing conditions, consistently accurate classification results were observed for all but one case with presumably borderline signal strength remaining after dilution.

Although there is no existing standard methodology to analytically validate an MCED test, the five studies presented above are consistent with several guidelines and perspectives, such as the United States Food & Drug Administration (FDA) and Clinical Laboratory & Standards Institute (CLSI) guidelines for validating multivariate and nucleic acid-based diagnostics [24, 25]. Specifically, FDA perspectives for assessing “complex signature” devices, defined as assays that integrate multiple variables into a biomarker signature to yield a single, patient-specific result (eg, cancer or non-cancer) [25], underscore the importance of establishing the reporting range and reproducibility of test results, as characterized by the input titration and reproducibility and repeatability studies. Similarly, CLSI guidelines for validation of nucleic acid assays highlight the importance of establishing test sensitivity and specificity and assessing the impact of potential interferents on test performance, which the analytical sensitivity and specificity, interferents, and cross-contamination studies addressed. In addition, the American Society of Clinical Oncology and the College of American Pathologists recommend that analytical validation of circulating tumor DNA assays include evaluation of not only laboratory procedures, but also bioinformatic analyses [13], such as the cross-contamination module. Nevertheless, there is a need for standardized guidelines and reference materials for evaluating multivariate cfDNA cancer diagnostics to support clinical development and use [11, 13, 26].

High analytical specificity is needed to achieve high clinical specificity (ie, a low false positive rate). In the present study, only 9 of 1204 non-cancer samples yielded a signal detected result with the MCED test. The reason for the occurrence of these false positives is not understood now and could be due to factors such as other biological conditions or undiscovered cancer. The fact that the majority of false positives represent independently repeated results with the same cancer signal origin arising from the same participant blood sample suggests that the signal detected is not due to analytical error. Investigation of such occurrences are part of the active development of the test.

The main strengths of this study are the analytical validation across the entire workflow of the MCED test and the large number of replicates in the five component studies. However, the study has several limitations. First, only a small number of cancers from the large number of cancers detected by the MCED test [17] were tested in each of the five studies. Given that the methylation-based methodology of the MCED test is tumor agnostic in terms of cancer detection, and analytical validation results were robust across all tumor stages of several representative cancers, such as breast, lung, and colorectal cancer, the study results are likely generalizable to other tumor types. Second, the validated results are limited to the range of tested parameters. This limitation is not likely to be clinically significant, as the upper bound of the tested ranges exceeded typical levels by several fold. For instance, in the study of interferents, the highest tested concentrations of bilirubin (20 mg/dL) and triglycerides (500 mg/dL) were >16× and >3× the upper limit of normal laboratory values (1.2 mg/dL and 150 mg/dL, respectively) [27]. Of note, even at these upper limits, MCED test results were not affected.

Finally, as an analytical validation, this study was not designed to evaluate clinical questions (eg, performance across different racial/ethnicity groups, positive predictive value, frequency of potentially confounding clinical conditions). The present study evaluated the feasibility of cancer signal detection by the MCED test in clinical samples from blood draws. The MCED test was applied to clinical samples of varying dilution, and results showed that the MCED test has the ability to detect cancer signals even when those signals are below levels that were present in the original samples. Clinical questions and test performance in target populations are being evaluated in other studies, including the prespecified, population-scale clinical validation study with a further refined assay and classifiers optimized for screening [18, 28].

In conclusion, this analytical validation study supports continued clinical development of a targeted methylation cfDNA MCED test in two ongoing, prospective clinical validation studies (STRIVE [NCT03085888] and SUMMIT [NCT03934866]) in intended-use populations. In addition, the prospective, interventional PATHFINDER study (NCT04241796) is returning test results to clinicians and, if cancer is detected, evaluating the extent of follow-up testing needed to achieve diagnostic resolution [19]. These studies are designed to provide a robust framework of evidence needed for implementing an MCED test in clinical practice.

## Supporting information

S1 Fig The multi-cancer early detection test uses a machine-learning classifier to call samples as cancer (asterisks) or non-cancer (open circles) based on a classification threshold of 0.994 (red dotted line). The percentile of a sample’s classifier-derived score among non-cancer samples in the training set, known as the binary classification score, is positively correlated with variant allele frequency (proportion of cell-free DNA fragments with variants identified in samples with matched tumor biopsy samples) in breast cancer (red), colorectal cancer (olive), head and neck cancer (green), and lung cancer (teal and magenta) samples.
(TIF)

S2 Fig *In silico* dilution analysis of additional early- and later-stage cancer cases. Each *in silico* titration sample was simulated by mixing a random fraction of the cancer sample reads with non-cancer sample reads. The ratio of cancer sample reads to non-cancer sample reads is referred to as the *in silico* titration level, and the reciprocal ratio is referred to as the fold dilution. For each cancer sample, 3 *in silico* titration samples were generated for each *in silico* titration level. The in silico LOD was expressed as the lowest *in silico* titration level (corresponds to the highest fold dilution) that a source sample could undergo and retain 100% classification accuracy across 3 *in silico* titration sample replicates. The *in silico* LODs were binned according to fold dilutions falling within 1–1.33x, 1.33-2x, 2-4x, and >4x. The frequency of detected samples by stage at each fold-dilution are shown.
(TIF)

S3 Fig In vitro dilution analysis of additional early-stage cases. cfDNA samples from seven cancer participants (3 stage I and 4 stage II) from the CCGA2 sub-cohort were diluted with non-cancer cfDNA from 43 unique non-cancer participants to generate 2–20 replicates (12 baseline non-cancer replicates also were processed). Replicates that had a cancer signal detected by the MCED test had a cancer score (1 minus the non-cancer score) of >0.685 (dotted vertical line).
(TIF)

S4 Fig Effect of various concentrations of 4 potential interferents on binary target coverage. Non-cancer plasma samples (red circles) and cancer admixtures (blue circles) were spiked with bilirubin (0–20 mg/dL), high-molecular-weight genomic DNA (0–200% of total cell-free DNA [cfDNA] extracted from unspiked samples), hemoglobin (0–2000 mg/dL), or triglycerides (0–500 mg/dL). Cancer admixtures were generated by adding abnormally methylated DNA from human HCT116 DKO cells to non-cancer plasma samples. Boxes indicate 25th and 75th percentiles and the line inside corresponds to the median. Whiskers extend to minimum and maximum values, excluding outliers. Data points have been slightly offset horizontally (jittered) to better visualize points that may otherwise overlap. gDNA, genomic DNA.
(TIF)

S1 Table Sample information for *in silico* dilution analysis. (DOCX)

S2 Table Level of detection hit rates. VAF, variant allele fraction.
(DOCX)

S3 Table Specificity of a methylation-based cell-free DNA multi-cancer early detection test in participants without cancer. CCGA, Circulating Cell-free DNA Genome Atlas study; CI, confidence interval.
(DOCX)
