# Supplementary figures and images for: Analytical validation of a multi-cancer early detection test with cancer signal origin using a cell-free DNA–based targeted methylation assay
Source: PLoS One. 2023 Apr 14;18(4):e0283001. doi: 10.1371/journal.pone.0283001 (PMC10104288; doi:10.1371/journal.pone.0283001)

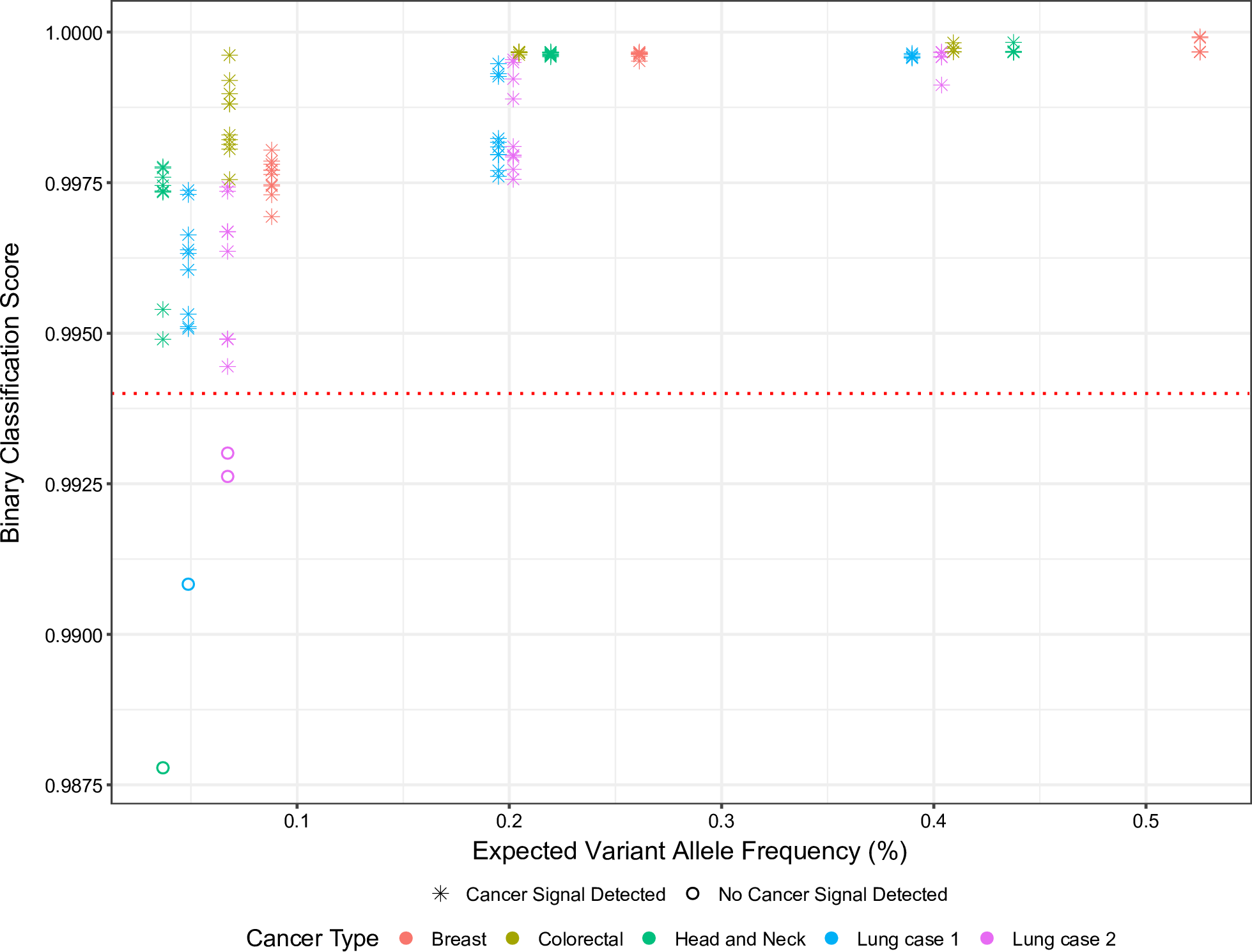

Supplement: S1 Fig — The percentile of a sample’s classifier-derived score among non-cancer samples in the training set, known as the binary classification score, is positively correlated with variant allele frequency (proportion of cell-free DNA fragments with variants identified in samples with matched tumor biopsy samples) in breast cancer (red), colorectal cancer (olive), head and neck cancer (green), and lung cancer (teal and magenta) samples. (TIF) [file pone.0283001.s001.tif]

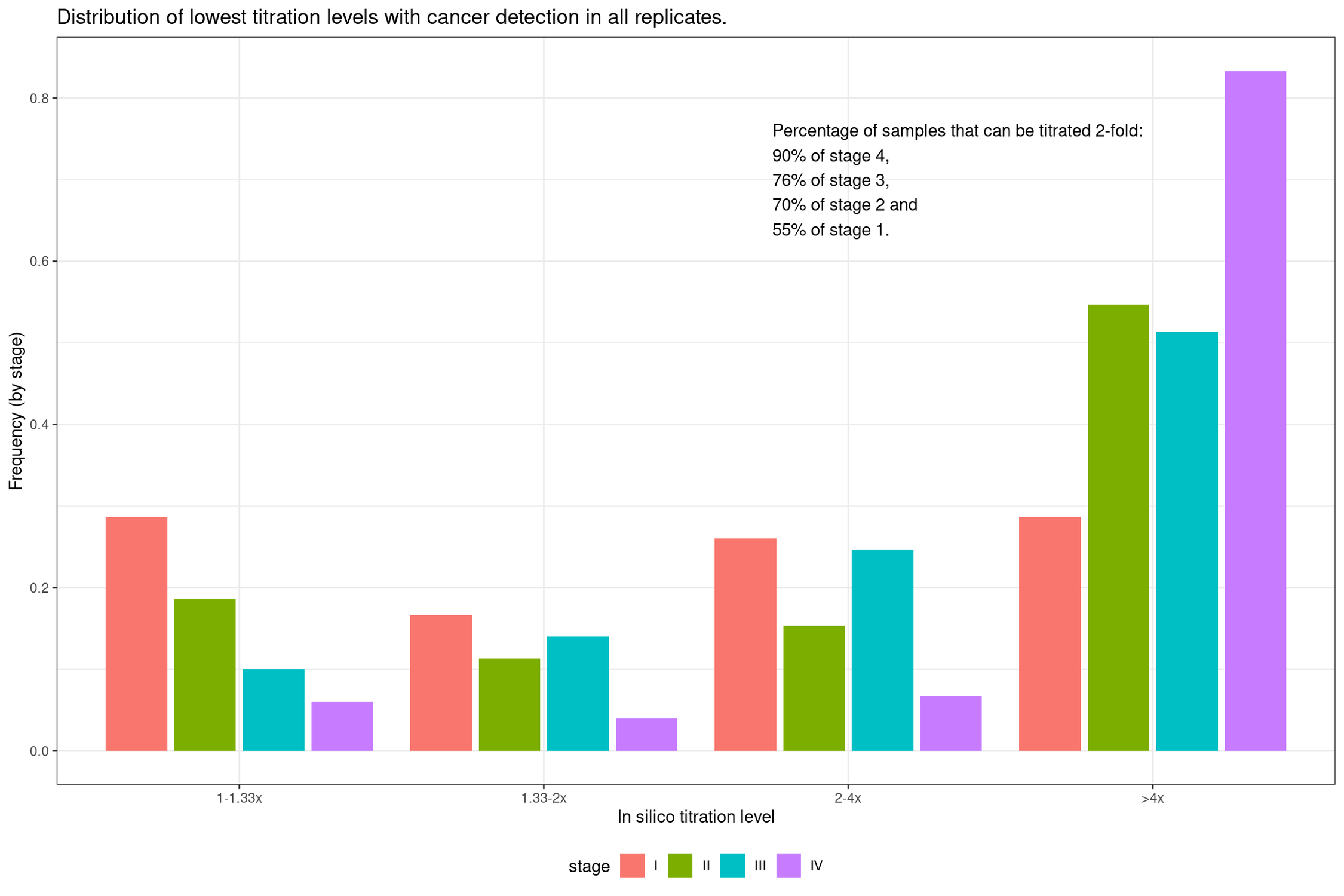

Supplement: S2 Fig — Each in silico titration sample was simulated by mixing a random fraction of the cancer sample reads with non-cancer sample reads. The ratio of cancer sample reads to non-cancer sample reads is referred to as the in silico titration level, and the reciprocal ratio is referred to as the fold dilution. For each cancer sample, 3 in silico titration samples were generated for each in silico titration level. The in silico LOD was expressed as the lowest in silico titration level (corresponds to the highest fold dilution) that a source sample could undergo and retain 100% classification accuracy across 3 in silico titration sample replicates. The in silico LODs were binned according to fold dilutions falling within 1–1.33x, 1.33-2x, 2-4x, and >4x. The frequency of detected samples by stage at each fold-dilution are shown. (TIF) [file pone.0283001.s002.tif]

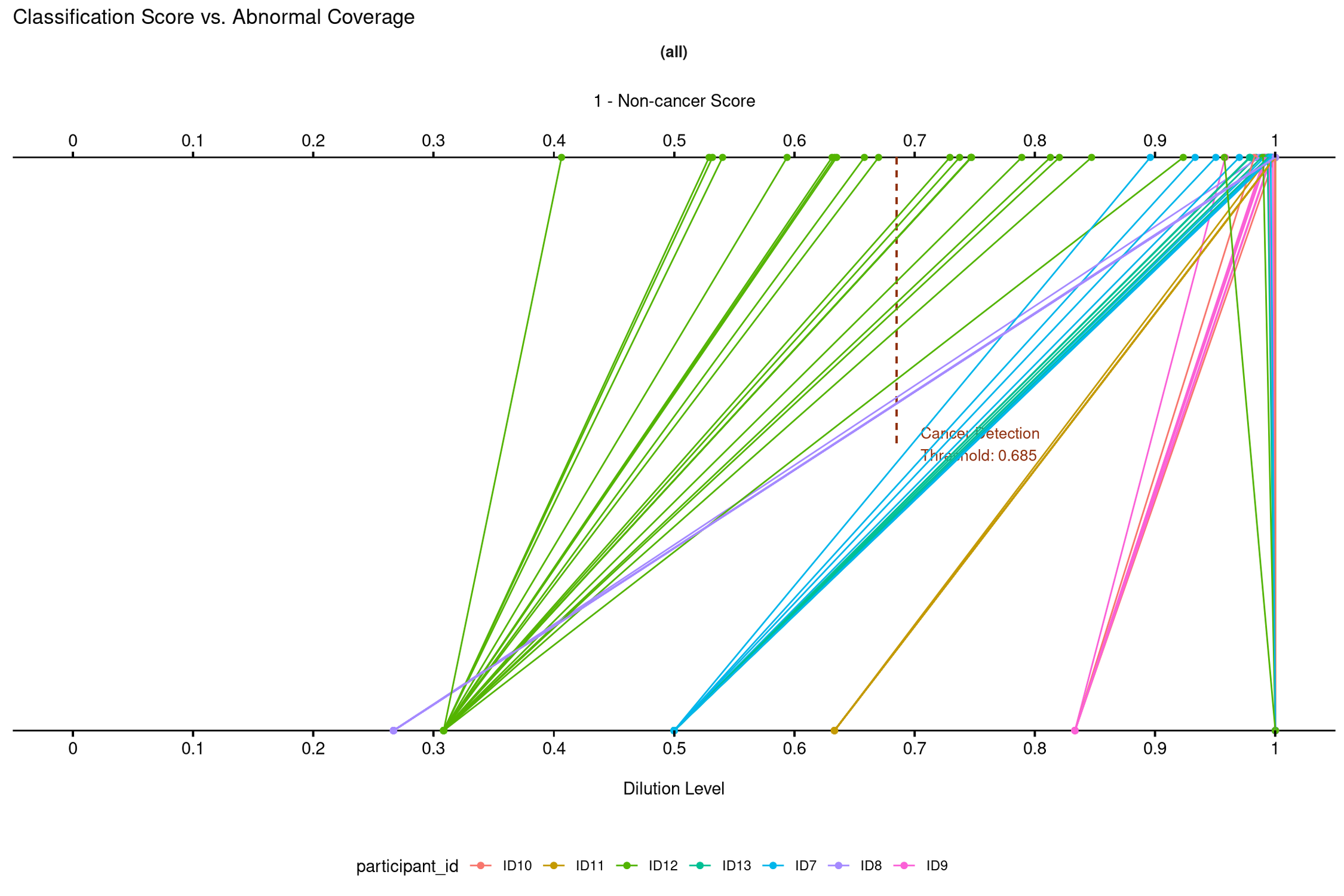

Supplement: S3 Fig — cfDNA samples from seven cancer participants (3 stage I and 4 stage II) from the CCGA2 sub-cohort were diluted with non-cancer cfDNA from 43 unique non-cancer participants to generate 2–20 replicates (12 baseline non-cancer replicates also were processed). Replicates that had a cancer signal detected by the MCED test had a cancer score (1 minus the non-cancer score) of >0.685 (dotted vertical line). (TIF) [file pone.0283001.s003.tif]

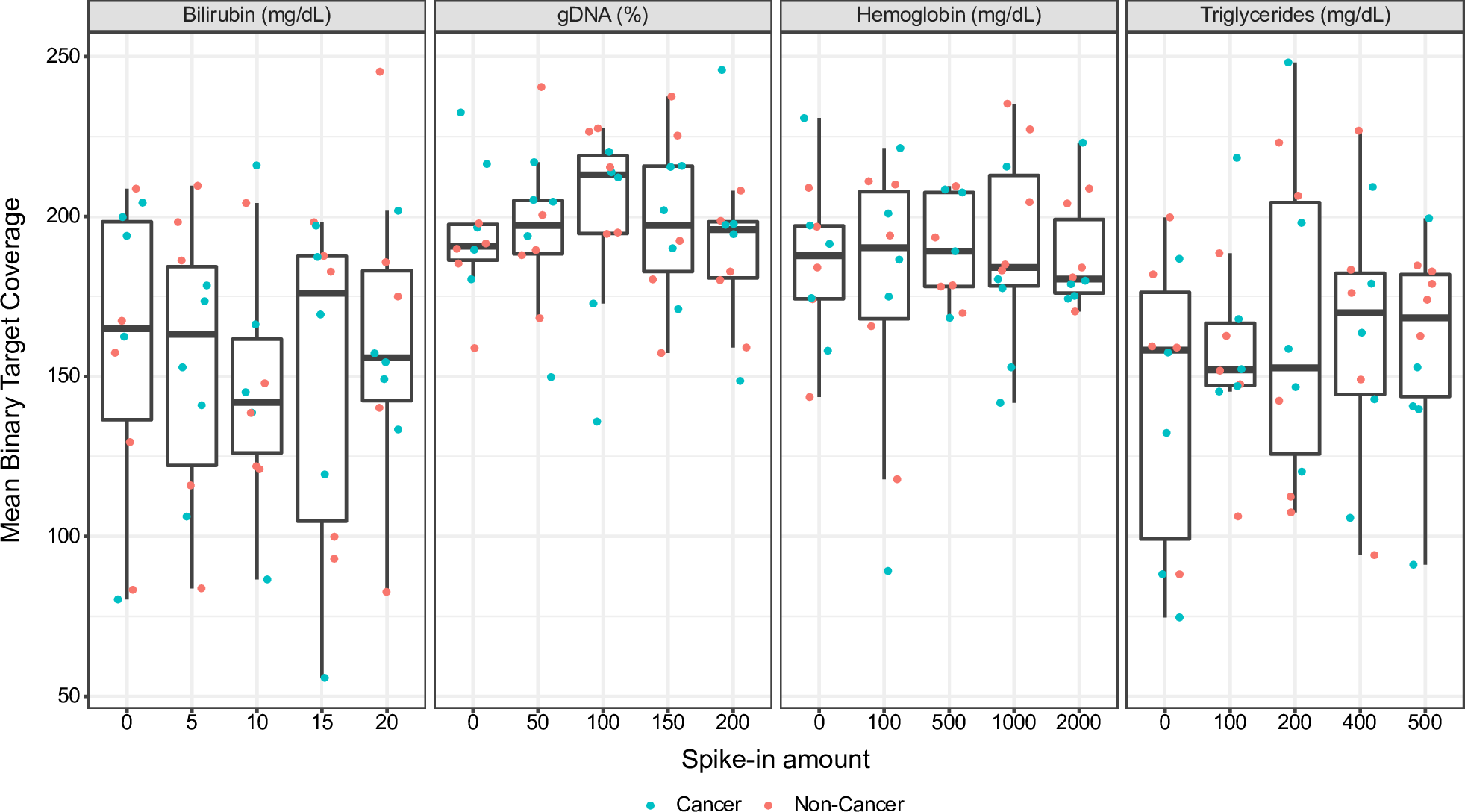

Supplement: S4 Fig — Non-cancer plasma samples (red circles) and cancer admixtures (blue circles) were spiked with bilirubin (0–20 mg/dL), high-molecular-weight genomic DNA (0–200% of total cell-free DNA [cfDNA] extracted from unspiked samples), hemoglobin (0–2000 mg/dL), or triglycerides (0–500 mg/dL). Cancer admixtures were generated by adding abnormally methylated DNA from human HCT116 DKO cells to non-cancer plasma samples. Boxes indicate 25th and 75th percentiles and the line inside corresponds to the median. Whiskers extend to minimum and maximum values, excluding outliers. Data points have been slightly offset horizontally (jittered) to better visualize points that may otherwise overlap. gDNA, genomic DNA. (TIF) [file pone.0283001.s004.tif]
