# Supplementary material for: Analytical validation of a multi-cancer early detection test with cancer signal origin using a cell-free DNA–based targeted methylation assay
Source: PLoS One. 2023 Apr 14;18(4):e0283001. doi: 10.1371/journal.pone.0283001 (PMC10104288; doi:10.1371/journal.pone.0283001)
Supplement: S1 Table — (DOCX) [file pone.0283001.s005.docx]

**S1 Table. Sample Information for *In Silico* Dilution Analysis**

| **Sample/Participant ID** | **Cancer Type** | **Clinical Stage** | **Fold Dilution** |
| --- | --- | --- | --- |
| ID55 | Pancreas | I | 1.5 |
| ID56 | Pancreas | I | 1.1 |
| ID57 | Plasma Cell Neoplasm | I | 3.4 |
| ID58 | Uterus | I | 1 |
| ID59 | Cervix | I | 27 |
| ID60 | Other | I | 2.4 |
| ID61 | Plasma Cell Neoplasm | I | 1.7 |
| ID62 | Uterus | I | 1 |
| ID63 | Breast | I | 1.4 |
| ID13 | Head and Neck | I | 4.6 |
| ID64 | Uterus | I | 2.3 |
| ID65 | Breast | I | 1.2 |
| ID66 | Ovary | I | 23.8 |
| ID67 | Head and Neck | I | 23.8 |
| ID68 | Uterus | I | 20.4 |
| ID7 | Colon Rectum | I | 3.7 |
| ID69 | Urothelial Tract | I | 4.5 |
| ID70 | Pancreas | I | 1 |
| ID71 | Colon Rectum | I | 1.2 |
| ID72 | Uterus | I | 1.3 |
| ID73 | Uterus | I | 5 |
| ID74 | Uterus | I | 1 |
| ID75 | Uterus | I | 2.9 |
| ID76 | Lung | I | 1.4 |
| ID77 | Cervix | I | 1 |
| ID78 | Lung | I | 83.3 |
| ID79 | Head and Neck | I | 76.9 |
| ID80 | Uterus | I | 2.2 |
| ID81 | Liver Bile duct | I | 1.1 |
| ID82 | Uterus | I | 2.2 |
| ID83 | Plasma Cell Neoplasm | I | 5.8 |
| ID84 | Breast | I | 1 |
| ID85 | Colon Rectum | I | 1.8 |
| ID86 | Ovary | I | 1 |
| ID87 | Pancreas | I | 1 |
| ID88 | Other | I | 3 |
| ID89 | Lung | I | 1.7 |
| ID90 | Plasma Cell Neoplasm | I | 4.4 |
| ID12 | Lung | I | 3.8 |
| ID91 | Lung | I | 1.1 |
| ID92 | Lung | I | 1.4 |
| ID93 | Uterus | I | 1.9 |
| ID94 | Plasma Cell Neoplasm | I | 32.3 |
| ID95 | Colon Rectum | I | 1 |
| ID96 | Head and Neck | I | 9.3 |
| ID97 | Bladder | I | 2.3 |
| ID98 | Stomach | I | 2 |
| ID99 | Lymphoma | I | 30.3 |
| ID100 | Uterus | I | 1.6 |
| ID101 | Pancreas | I | 1.8 |
| ID102 | Head and Neck | I | 7.1 |
| ID103 | Pancreas | I | 5 |
| ID104 | Lung | I | 2.4 |
| ID105 | Sarcoma | I | 166.7 |
| ID106 | Colon Rectum | I | 1.4 |
| ID107 | Lymphoma | I | 333.3 |
| ID108 | Bladder | I | 2.7 |
| ID109 | Lung | I | 2.3 |
| ID32 | Anus | I | 10.4 |
| ID110 | Head and Neck | I | 16.7 |
| ID111 | Uterus | I | 1.5 |
| ID112 | Uterus | I | 1.8 |
| ID113 | Breast | I | 1 |
| ID114 | Other | I | 7 |
| ID115 | Lung | I | 1.2 |
| ID116 | Bladder | I | 1 |
| ID32 | Anus | I | 7 |
| ID117 | Plasma Cell Neoplasm | I | 4.9 |
| ID118 | Uterus | I | 1 |
| ID119 | Lymphoma | I | 16.7 |
| ID120 | Pancreas | I | 5.7 |
| ID121 | Head and Neck | I | 7 |
| ID122 | Uterus | I | 1.2 |
| ID123 | Lung | I | 2.1 |
| ID124 | Colon Rectum | I | 2.2 |
| ID125 | Liver Bile duct | I | 20 |
| ID126 | Uterus | I | 1.5 |
| ID127 | Anus | I | 1.3 |
| ID128 | Lung | I | 4.1 |
| ID129 | Esophagus | I | 1.5 |
| ID130 | Liver Bile duct | I | 7 |
| ID131 | Lung | I | 71.4 |
| ID132 | Head and Neck | I | 22.2 |
| ID133 | Uterus | I | 1 |
| ID12 | Lung | I | 2.3 |
| ID134 | Uterus | I | 1.2 |
| ID135 | Liver Bile duct | I | 16.9 |
| ID136 | Pancreas | I | 1.1 |
| ID137 | Uterus | I | 1.5 |
| ID138 | Uterus | I | 1 |
| ID139 | Lung | I | 2.1 |
| ID140 | Breast | I | 2.7 |
| ID141 | Colon Rectum | I | 1.6 |
| ID142 | Ovary | I | 1 |
| ID13 | Head and Neck | I | 3.2 |
| ID143 | Uterus | I | 2.4 |
| ID144 | Liver Bile duct | I | 15.4 |
| ID145 | Bladder | I | 1 |
| ID146 | Pancreas | I | 1 |
| ID147 | Pancreas | I | 1 |
| ID148 | Urothelial Tract | I | 2.2 |
| ID7 | Colon Rectum | I | 3.4 |
| ID149 | Uterus | I | 1.5 |
| ID150 | Lung | I | 1.8 |
| ID151 | Pancreas | I | 1 |
| ID152 | Lung | I | 1.1 |
| ID153 | Other | I | 3.2 |
| ID154 | Lymphoma | I | 1 |
| ID155 | Other | I | 2.4 |
| ID156 | Breast | I | 1.2 |
| ID157 | Uterus | I | 3.5 |
| ID158 | Liver Bile duct | I | 2.6 |
| ID159 | Colon Rectum | I | 2.1 |
| ID160 | Uterus | I | 1 |
| ID161 | Pancreas | I | 1.4 |
| ID162 | Other | I | 3 |
| ID163 | Pancreas | I | 1.7 |
| ID164 | Head and Neck | I | 71.4 |
| ID165 | Uterus | I | 3.2 |
| ID166 | Sarcoma | I | 1 |
| ID167 | Breast | I | 1 |
| ID168 | Uterus | I | 1 |
| ID169 | Lung | I | 1.1 |
| ID170 | Uterus | I | 3.1 |
| ID171 | Bladder | I | 2.9 |
| ID172 | Esophagus | I | 3.2 |
| ID173 | Plasma Cell Neoplasm | I | 3.9 |
| ID174 | Colon Rectum | I | 1.6 |
| ID175 | Lung | I | 7.5 |
| ID176 | Ovary | I | 26.3 |
| ID177 | Anus | I | 2.3 |
| ID178 | Plasma Cell Neoplasm | I | 6.8 |
| ID179 | Sarcoma | I | 200 |
| ID180 | Pancreas | I | 4.9 |
| ID181 | Lymphoma | I | 2.2 |
| ID182 | Breast | I | 3.4 |
| ID183 | Colon Rectum | I | 2.6 |
| ID184 | Plasma Cell Neoplasm | I | 1.8 |
| ID185 | Uterus | I | 1.3 |
| ID186 | Stomach | I | 1.2 |
| ID187 | Lung | I | 3.6 |
| ID188 | Head and Neck | I | 23.3 |
| ID189 | Lung | I | 1.8 |
| ID190 | Cervix | I | 1.2 |
| ID191 | Colon Rectum | I | 2 |
| ID192 | Head and Neck | I | 7.9 |
| ID193 | Uterus | I | 1 |
| ID194 | Plasma Cell Neoplasm | I | 32.3 |
| ID195 | Uterus | I | 24.4 |
| ID196 | Breast | I | 1.1 |
| ID197 | Colon Rectum | II | 1 |
| ID198 | Breast | II | 100 |
| ID199 | Lung | II | 5.1 |
| ID200 | Lung | II | 200 |
| ID201 | Lung | II | 3.2 |
| ID202 | Breast | II | 15.4 |
| ID203 | Esophagus | II | 3.6 |
| ID204 | Breast | II | 5.3 |
| ID205 | Head and Neck | II | 38.5 |
| ID206 | Liver Bile duct | II | 29.4 |
| ID207 | Breast | II | 5.9 |
| ID208 | Colon Rectum | II | 27 |
| ID209 | Breast | II | 2.4 |
| ID210 | Breast | II | 4.8 |
| ID211 | Stomach | II | 1.1 |
| ID212 | Esophagus | II | 1.2 |
| ID213 | Prostate | II | 1.4 |
| ID30 | Head and Neck | II | 250 |
| ID214 | Plasma Cell Neoplasm | II | 1.4 |
| ID215 | Bladder | II | 3.6 |
| ID216 | Plasma Cell Neoplasm | II | 6.3 |
| ID217 | Plasma Cell Neoplasm | II | 1.9 |
| ID218 | Pancreas | II | 17.2 |
| ID219 | Head and Neck | II | 43.5 |
| ID33 | Breast | II | 125 |
| ID220 | Lung | II | 1.1 |
| ID221 | Breast | II | 1.3 |
| ID222 | Urothelial Tract | II | 1 |
| ID223 | Liver Bile duct | II | 27.8 |
| ID44 | Breast | II | 2.6 |
| ID224 | Lymphoma | II | 83.3 |
| ID225 | Breast | II | 17.5 |
| ID226 | Stomach | II | 1.8 |
| ID227 | Colon Rectum | II | 1.6 |
| ID228 | Lymphoma | II | 111.1 |
| ID229 | Breast | II | 3 |
| ID230 | Breast | II | 4 |
| ID231 | Breast | II | 7.9 |
| ID232 | Cervix | II | 45.5 |
| ID50 | Liver Bile duct | II | 250 |
| ID233 | Uterus | II | 1 |
| ID234 | Breast | II | 1 |
| ID235 | Colon Rectum | II | 1 |
| ID236 | Breast | II | 1 |
| ID237 | Colon Rectum | II | 1.9 |
| ID238 | Breast | II | 1.7 |
| ID29 | Pancreas | II | 6.1 |
| ID239 | Breast | II | 1.5 |
| ID240 | Lung | II | 4.3 |
| ID241 | Colon Rectum | II | 3.9 |
| ID242 | Lymphoma | II | 9.3 |
| ID243 | Colon Rectum | II | 3 |
| ID244 | Breast | II | 1.3 |
| ID245 | Lymphoma | II | 111.1 |
| ID246 | Breast | II | 5.4 |
| ID247 | Plasma Cell Neoplasm | II | 13.2 |
| ID248 | Liver Bile duct | II | 166.7 |
| ID20 | Head and Neck | II | 7.4 |
| ID249 | Breast | II | 1.5 |
| ID250 | Colon Rectum | II | 5 |
| ID251 | Esophagus | II | 2.6 |
| ID252 | Breast | II | 1.5 |
| ID253 | Prostate | II | 1 |
| ID254 | Plasma Cell Neoplasm | II | 14.9 |
| ID255 | Lymphoma | II | 2.2 |
| ID256 | Head and Neck | II | 17.2 |
| ID257 | Colon Rectum | II | 1.5 |
| ID258 | Head and Neck | II | 10.4 |
| ID10 | Esophagus | II | 3.3 |
| ID259 | Lung | II | 1 |
| ID260 | Prostate | II | 14.1 |
| ID261 | Lung | II | 200 |
| ID262 | Prostate | II | 8.1 |
| ID263 | Pancreas | II | 10.1 |
| ID264 | Anus | II | 5.2 |
| ID11 | Lung | II | 6.9 |
| ID265 | Breast | II | 6.7 |
| ID266 | Breast | II | 71.4 |
| ID267 | Breast | II | 7.2 |
| ID268 | Colon Rectum | II | 3.2 |
| ID269 | Lymphoma | II | 90.9 |
| ID270 | Colon Rectum | II | 1.4 |
| ID271 | Colon Rectum | II | 1.6 |
| ID272 | Head and Neck | II | 7.2 |
| ID273 | Breast | II | 9.6 |
| ID274 | Lymphoma | II | 1 |
| ID275 | Lung | II | 1.8 |
| ID276 | Breast | II | 2.2 |
| ID277 | Lymphoma | II | 76.9 |
| ID8 | Pancreas | II | 9.1 |
| ID278 | Lung | II | 6.8 |
| ID279 | Head and Neck | II | 1 |
| ID280 | Cervix | II | 40 |
| ID281 | Lymphoma | II | 1 |
| ID282 | Breast | II | 6.3 |
| ID283 | Plasma Cell Neoplasm | II | 1 |
| ID284 | Breast | II | 1 |
| ID285 | Breast | II | 62.5 |
| ID286 | Prostate | II | 1 |
| ID287 | Lung | II | 15.4 |
| ID9 | Head and Neck | II | 5.3 |
| ID288 | Breast | II | 19.2 |
| ID289 | Lymphoma | II | 2.2 |
| ID290 | Lymphoma | II | 76.9 |
| ID291 | Lung | II | 1.2 |
| ID292 | Lymphoma | II | 90.9 |
| ID293 | Breast | II | 2.2 |
| ID294 | Lymphoma | II | 333.3 |
| ID295 | Breast | II | 6.3 |
| ID296 | Lymphoma | II | 125 |
| ID297 | Breast | II | 4.9 |
| ID298 | Esophagus | II | 1.3 |
| ID299 | Pancreas | II | 2.5 |
| ID300 | Breast | II | 6 |
| ID301 | Prostate | II | 1 |
| ID302 | Stomach | II | 1 |
| ID303 | Breast | II | 22.2 |
| ID304 | Other | II | 11.5 |
| ID305 | Lung | II | 111.1 |
| ID306 | Other | II | 1.7 |
| ID307 | Prostate | II | 1 |
| ID308 | Breast | II | 3.1 |
| ID309 | Colon Rectum | II | 5.2 |
| ID310 | Plasma Cell Neoplasm | II | 111.1 |
| ID311 | Colon Rectum | II | 4.9 |
| ID312 | Head and Neck | II | 52.6 |
| ID313 | Colon Rectum | II | 2.7 |
| ID314 | Colon Rectum | II | 27 |
| ID315 | Lung | II | 3.8 |
| ID316 | Liver Bile duct | II | 125 |
| ID317 | Breast | II | 2.8 |
| ID318 | Plasma Cell Neoplasm | II | 18.9 |
| ID319 | Pancreas | II | 4.9 |
| ID320 | Breast | II | 1.6 |
| ID321 | Anus | II | 9.6 |
| ID44 | Breast | II | 4.8 |
| ID322 | Head and Neck | II | 12.8 |
| ID323 | Breast | II | 3.6 |
| ID324 | Lymphoma | II | 1 |
| ID325 | Plasma Cell Neoplasm | II | 10.9 |
| ID326 | Pancreas | II | 2.3 |
| ID327 | Colon Rectum | II | 1.9 |
| ID328 | Breast | II | 1.3 |
| ID329 | Breast | II | 3.9 |
| ID330 | Bladder | II | 4.9 |
| ID331 | Colon Rectum | II | 1 |
| ID332 | Plasma Cell Neoplasm | II | 43.5 |
| ID333 | Urothelial Tract | II | 22.2 |
| ID334 | Head and Neck | II | 47.6 |
| ID335 | Breast | II | 1 |
| ID336 | Colon Rectum | III | 34.5 |
| ID337 | Lymphoma | III | 1 |
| ID338 | Head and Neck | III | 1.6 |
| ID339 | Anus | III | 3.8 |
| ID340 | Ovary | III | 1.2 |
| ID341 | Lung | III | 500 |
| ID342 | Head and Neck | III | 125 |
| ID343 | Pancreas | III | 3 |
| ID344 | Other | III | 19.6 |
| ID345 | Lung | III | 3.6 |
| ID346 | Ovary | III | 5.6 |
| ID25 | Esophagus | III | 17.2 |
| ID347 | Lung | III | 62.5 |
| ID348 | Lung | III | 21.3 |
| ID349 | Breast | III | 24.4 |
| ID350 | Ovary | III | 111.1 |
| ID351 | Lymphoma | III | 3.2 |
| ID352 | Ovary | III | 7.2 |
| ID353 | Lung | III | 90.9 |
| ID354 | Cervix | III | 19.2 |
| ID27 | Lung | III | 1000 |
| ID355 | Lymphoma | III | 2.1 |
| ID356 | Pancreas | III | 3.3 |
| ID357 | Lung | III | 12.5 |
| ID358 | Plasma Cell Neoplasm | III | 43.5 |
| ID359 | Head and Neck | III | 2.3 |
| ID360 | Lymphoma | III | 1 |
| ID361 | Colon Rectum | III | 1.4 |
| ID36 | Plasma Cell Neoplasm | III | 12.7 |
| ID362 | Ovary | III | 1 |
| ID363 | Cervix | III | 2 |
| ID364 | Head and Neck | III | 2.7 |
| ID365 | Stomach | III | 19.6 |
| ID366 | Lung | III | 5 |
| ID367 | Other | III | 14.9 |
| ID368 | Colon Rectum | III | 200 |
| ID369 | Lung | III | 2.9 |
| ID370 | Pancreas | III | 9.5 |
| ID46 | Ovary | III | 1.8 |
| ID371 | Other | III | 9.6 |
| ID372 | Lymphoma | III | 66.7 |
| ID24 | Lung | III | 11 |
| ID373 | Breast | III | 12.8 |
| ID374 | Lung | III | 20 |
| ID375 | Esophagus | III | 1.6 |
| ID376 | Pancreas | III | 2.1 |
| ID377 | Liver Bile duct | III | 166.7 |
| ID378 | Ovary | III | 111.1 |
| ID379 | Lung | III | 2.6 |
| ID380 | Breast | III | 5.9 |
| ID381 | Lung | III | 34.5 |
| ID382 | Liver Bile duct | III | 8 |
| ID383 | Head and Neck | III | 1 |
| ID384 | Gallbladder | III | 3.1 |
| ID385 | Kidney | III | 2.3 |
| ID386 | Head and Neck | III | 21.7 |
| ID387 | Esophagus | III | 2 |
| ID388 | Colon Rectum | III | 15.6 |
| ID389 | Colon Rectum | III | 1.8 |
| ID390 | Colon Rectum | III | 1.8 |
| ID391 | Breast | III | 8.7 |
| ID392 | Ovary | III | 9.1 |
| ID393 | Cervix | III | 1.5 |
| ID394 | Breast | III | 166.7 |
| ID395 | Breast | III | 1.2 |
| ID24 | Lung | III | 9.3 |
| ID396 | Pancreas | III | 1 |
| ID397 | Lymphoma | III | 27.8 |
| ID398 | Breast | III | 1.4 |
| ID399 | Lymphoma | III | 1 |
| ID400 | Uterus | III | 1.5 |
| ID401 | Lung | III | 1.8 |
| ID402 | Lymphoma | III | 142.9 |
| ID403 | Colon Rectum | III | 11.6 |
| ID404 | Ovary | III | 13.7 |
| ID405 | Breast | III | 2.4 |
| ID406 | Ovary | III | 16.7 |
| ID407 | Kidney | III | 2.6 |
| ID408 | Plasma Cell Neoplasm | III | 2.2 |
| ID409 | Breast | III | 10.6 |
| ID410 | Colon Rectum | III | 7.8 |
| ID411 | Prostate | III | 3.5 |
| ID412 | Lung | III | 3.2 |
| ID413 | Kidney | III | 4.6 |
| ID414 | Pancreas | III | 2.7 |
| ID415 | Plasma Cell Neoplasm | III | 30.3 |
| ID416 | Other | III | 11.1 |
| ID417 | Colon Rectum | III | 2.8 |
| ID418 | Lung | III | 1.6 |
| ID419 | Lung | III | 125 |
| ID420 | Lung | III | 2.6 |
| ID421 | Breast | III | 62.5 |
| ID422 | Esophagus | III | 1.4 |
| ID423 | Ovary | III | 1 |
| ID424 | Lung | III | 55.6 |
| ID425 | Pancreas | III | 3.3 |
| ID426 | Lymphoma | III | 5.4 |
| ID427 | Lung | III | 6.2 |
| ID428 | Lung | III | 2.9 |
| ID429 | Breast | III | 1.1 |
| ID430 | Breast | III | 1.8 |
| ID431 | Lung | III | 166.7 |
| ID432 | Lung | III | 1.6 |
| ID433 | Liver Bile duct | III | 52.6 |
| ID434 | Anus | III | 2.1 |
| ID435 | Ovary | III | 50 |
| ID436 | Breast | III | 200 |
| ID437 | Breast | III | 8.1 |
| ID438 | Lung | III | 35.7 |
| ID439 | Lung | III | 1.8 |
| ID440 | Head and Neck | III | 1.4 |
| ID441 | Lung | III | 1.3 |
| ID442 | Colon Rectum | III | 1 |
| ID443 | Colon Rectum | III | 5.1 |
| ID444 | Colon Rectum | III | 1.7 |
| ID445 | Ovary | III | 18.2 |
| ID446 | Lung | III | 83.3 |
| ID447 | Ovary | III | 166.7 |
| ID448 | Lung | III | 12.2 |
| ID449 | Head and Neck | III | 4.8 |
| ID450 | Lung | III | 250 |
| ID451 | Esophagus | III | 2.6 |
| ID452 | Esophagus | III | 2.4 |
| ID453 | Kidney | III | 3.3 |
| ID454 | Colon Rectum | III | 2.8 |
| ID455 | Plasma Cell Neoplasm | III | 11.5 |
| ID456 | Lung | III | 47.6 |
| ID457 | Pancreas | III | 1.3 |
| ID458 | Head and Neck | III | 1.2 |
| ID459 | Colon Rectum | III | 8.2 |
| ID460 | Ovary | III | 8.1 |
| ID461 | Pancreas | III | 3.9 |
| ID462 | Lymphoma | III | 111.1 |
| ID463 | Lung | III | 3.4 |
| ID464 | Lymphoma | III | 1 |
| ID465 | Esophagus | III | 3.7 |
| ID39 | Plasma Cell Neoplasm | III | 200 |
| ID466 | Plasma Cell Neoplasm | III | 2.6 |
| ID467 | Stomach | III | 52.6 |
| ID468 | Lung | III | 111.1 |
| ID469 | Breast | III | 2.7 |
| ID470 | Head and Neck | III | 1.7 |
| ID471 | Gallbladder | III | 27 |
| ID472 | Ovary | III | 7.1 |
| ID473 | Lymphoma | III | 1 |
| ID474 | Pancreas | III | 10.1 |
| ID475 | Lung | III | 2.7 |
| ID476 | Colon Rectum | III | 3.1 |
| ID477 | Lung | III | 3.7 |
| ID478 | Pancreas | III | 3.4 |
| ID479 | Head and Neck | IV | 6.9 |
| ID480 | Lung | IV | 9.1 |
| ID481 | Lung | IV | 21.3 |
| ID482 | Esophagus | IV | 2.5 |
| ID483 | Liver Bile duct | IV | 33.3 |
| ID484 | Lung | IV | 166.7 |
| ID485 | Liver Bile duct | IV | 333.3 |
| ID486 | Colon Rectum | IV | 333.3 |
| ID487 | Lung | IV | 55.6 |
| ID488 | Lung | IV | 12.3 |
| ID489 | Lung | IV | 4 |
| ID490 | Lung | IV | 333.3 |
| ID491 | Lung | IV | 5.2 |
| ID492 | Lung | IV | 250 |
| ID493 | Colon Rectum | IV | 333.3 |
| ID494 | Head and Neck | IV | 100 |
| ID495 | Lung | IV | 4 |
| ID496 | Lung | IV | 41.7 |
| ID497 | Gallbladder | IV | 6.9 |
| ID498 | Lymphoma | IV | 2.9 |
| ID499 | Colon Rectum | IV | 12.7 |
| ID500 | Liver Bile duct | IV | 166.7 |
| ID501 | Esophagus | IV | 1.1 |
| ID502 | Colon Rectum | IV | 4.7 |
| ID503 | Head and Neck | IV | 16.1 |
| ID504 | Colon Rectum | IV | 200 |
| ID505 | Lung | IV | 32.3 |
| ID506 | Lung | IV | 1.5 |
| ID507 | Kidney | IV | 76.9 |
| ID508 | Lung | IV | 111.1 |
| ID509 | Pancreas | IV | 2.2 |
| ID510 | Lymphoma | IV | 30.3 |
| ID511 | Esophagus | IV | 76.9 |
| ID512 | Lymphoma | IV | 26.3 |
| ID513 | Head and Neck | IV | 1 |
| ID514 | Lymphoma | IV | 83.3 |
| ID515 | Stomach | IV | 250 |
| ID516 | Other | IV | 5.3 |
| ID517 | Head and Neck | IV | 200 |
| ID518 | Lung | IV | 25.6 |
| ID519 | Other | IV | 10.8 |
| ID520 | Breast | IV | 1.3 |
| ID521 | Lung | IV | 11.6 |
| ID522 | Pancreas | IV | 41.7 |
| ID523 | Lung | IV | 1 |
| ID524 | Prostate | IV | 90.9 |
| ID525 | Colon Rectum | IV | 35.7 |
| ID526 | Lung | IV | 5.2 |
| ID527 | Lung | IV | 25.6 |
| ID528 | Colon Rectum | IV | 333.3 |
| ID529 | Lung | IV | 1.4 |
| ID530 | Lung | IV | 47.6 |
| ID531 | Lung | IV | 7.7 |
| ID532 | Pancreas | IV | 1 |
| ID533 | Lung | IV | 1.4 |
| ID534 | Lung | IV | 21.7 |
| ID535 | Esophagus | IV | 3.9 |
| ID536 | Pancreas | IV | 125 |
| ID537 | Lung | IV | 1 |
| ID538 | Colon Rectum | IV | 66.7 |
| ID539 | Esophagus | IV | 9.8 |
| ID540 | Kidney | IV | 10.2 |
| ID541 | Lung | IV | 10.5 |
| ID542 | Breast | IV | 13 |
| ID543 | Kidney | IV | 10 |
| ID544 | Lung | IV | 16.4 |
| ID545 | Lung | IV | 500 |
| ID546 | Lung | IV | 13.9 |
| ID547 | Gallbladder | IV | 13 |
| ID548 | Pancreas | IV | 16.7 |
| ID549 | Liver Bile duct | IV | 41.7 |
| ID550 | Pancreas | IV | 71.4 |
| ID551 | Lung | IV | 71.4 |
| ID552 | Pancreas | IV | 41.7 |
| ID553 | Gallbladder | IV | 34.5 |
| ID554 | Lymphoma | IV | 1 |
| ID555 | Lung | IV | 83.3 |
| ID556 | Head and Neck | IV | 6 |
| ID557 | Lung | IV | 5.5 |
| ID558 | Colon Rectum | IV | 7.6 |
| ID559 | Pancreas | IV | 27.8 |
| ID560 | Lymphoma | IV | 13.3 |
| ID561 | Lung | IV | 26.3 |
| ID562 | Colon Rectum | IV | 250 |
| ID563 | Liver Bile duct | IV | 125 |
| ID564 | Pancreas | IV | 90.9 |
| ID565 | Head and Neck | IV | 19.2 |
| ID566 | Head and Neck | IV | 28.6 |
| ID567 | Lung | IV | 24.4 |
| ID568 | Pancreas | IV | 111.1 |
| ID569 | Esophagus | IV | 2.9 |
| ID570 | Head and Neck | IV | 14.9 |
| ID571 | Lung | IV | 250 |
| ID572 | Lymphoma | IV | 1000 |
| ID573 | Lung | IV | 1.1 |
| ID574 | Esophagus | IV | 1.3 |
| ID575 | Colon Rectum | IV | 4.8 |
| ID576 | Lung | IV | 62.5 |
| ID577 | Colon Rectum | IV | 200 |
| ID578 | Prostate | IV | 1.4 |
| ID579 | Lung | IV | 34.5 |
| ID580 | Lung | IV | 125 |
| ID581 | Breast | IV | 2.4 |
| ID582 | Esophagus | IV | 333.3 |
| ID583 | Lymphoma | IV | 50 |
| ID584 | Ovary | IV | 3.4 |
| ID585 | Stomach | IV | 83.3 |
| ID586 | Prostate | IV | 166.7 |
| ID587 | Esophagus | IV | 200 |
| ID588 | Pancreas | IV | 166.7 |
| ID589 | Lung | IV | 28.6 |
| ID590 | Prostate | IV | 16.4 |
| ID591 | Lymphoma | IV | 20.8 |
| ID592 | Head and Neck | IV | 15.6 |
| ID593 | Colon Rectum | IV | 20 |
| ID594 | Colon Rectum | IV | 500 |
| ID595 | Lung | IV | 11.2 |
| ID596 | Lung | IV | 200 |
| ID597 | Lymphoma | IV | 142.9 |
| ID598 | Lung | IV | 6.4 |
| ID599 | Prostate | IV | 2.2 |
| ID600 | Liver Bile duct | IV | 166.7 |
| ID601 | Esophagus | IV | 500 |
| ID602 | Esophagus | IV | 29.4 |
| ID603 | Esophagus | IV | 6.2 |
| ID604 | Ovary | IV | 27 |
| ID605 | Colon Rectum | IV | 500 |
| ID606 | Lymphoma | IV | 111.1 |
| ID607 | Pancreas | IV | 500 |
| ID608 | Pancreas | IV | 9.9 |
| ID609 | Lymphoma | IV | 2.2 |
| ID610 | Lung | IV | 24.4 |
| ID611 | Lung | IV | 24.4 |
| ID612 | Ovary | IV | 111.1 |
| ID613 | Prostate | IV | 31.2 |
| ID614 | Colon Rectum | IV | 5.6 |
| ID615 | Lung | IV | 43.5 |
| ID616 | Lung | IV | 166.7 |
| ID617 | Melanoma | IV | 333.3 |
| ID618 | Kidney | IV | 55.6 |
| ID619 | Pancreas | IV | 125 |
| ID620 | Kidney | IV | 1.4 |
| ID621 | Pancreas | IV | 125 |
| ID622 | Esophagus | IV | 1.9 |
| ID623 | Lung | IV | 8.2 |
| ID624 | Lung | IV | 250 |
| ID625 | Lymphoma | IV | 1000 |
| ID626 | Colon Rectum | IV | 76.9 |
| ID627 | Prostate | IV | 90.9 |
| ID628 | Lung | IV | 8.5 |
