# Supplementary material for: Analytical validation of a multi-cancer early detection test with cancer signal origin using a cell-free DNA–based targeted methylation assay
Source: PLoS One. 2023 Apr 14;18(4):e0283001. doi: 10.1371/journal.pone.0283001 (PMC10104288; doi:10.1371/journal.pone.0283001)
Supplement: S2 Table — VAF, variant allele fraction. (DOCX) [file pone.0283001.s006.docx]

**S2 Table. Level of Detection Hit Rates.**

| **Cancer Type** | **Tumor Fraction Level** | **Targeted Expected VAF** | **Total number Detected** | **Total Number in Group, n** | **Hit Rate**  **(total detected/n)** |
| --- | --- | --- | --- | --- | --- |
| Breast | High | 0.5254 | 5 | 5 | 1 |
| Breast | Low | 0.08804 | 10 | 10 | 1 |
| Breast | Mid | 0.26128 | 10 | 10 | 1 |
| Colorectal | High | 0.4092 | 5 | 5 | 1 |
| Colorectal | Low | 0.0682 | 10 | 10 | 1 |
| Colorectal | Mid | 0.2046 | 10 | 10 | 1 |
| Head and neck | High | 0.437562 | 5 | 5 | 1 |
| Head and neck | Low | 0.036792 | 9 | 10 | 0.9 |
| Head and neck | Mid | 0.219438 | 10 | 10 | 1 |
| Lung case 1 | High | 0.38976 | 5 | 5 | 1 |
| Lung case 1 | Low | 0.04872 | 9 | 10 | 0.9 |
| Lung case 1 | Mid | 0.19488 | 10 | 10 | 1 |
| Lung case 2 | High | 0.403704 | 5 | 5 | 1 |
| Lung case 2 | Low | 0.067284 | 8 | 10 | 0.8 |
| Lung case 2 | Mid | 0.201852 | 10 | 10 | 1 |
| Lymphoid neoplasm | High | 1.0824 | 5 | 5 | 1 |
| Lymphoid neoplasm | Low | 0.2706 | 5 | 10 | 0.5 |
| Lymphoid neoplasm | Mid | 0.5412 | 10 | 10 | 1 |
| Non-cancer | None | 0 | 0 | 24 | 0 |

VAF, variant allele fraction.
