# Supplementary material for: Analytical validation of a multi-cancer early detection test with cancer signal origin using a cell-free DNA–based targeted methylation assay
Source: PLoS One. 2023 Apr 14;18(4):e0283001. doi: 10.1371/journal.pone.0283001 (PMC10104288; doi:10.1371/journal.pone.0283001)
Supplement: S3 Table — CCGA, Circulating Cell-free DNA Genome Atlas study; CI, confidence interval. (DOCX) [file pone.0283001.s007.docx]

**S3 Table. Specificity of a Methylation-Based Cell-Free DNA Multi-cancer Early Detection Test in Participants Without Cancer**

| **Source** | **Participants (n)** | **Samples (n)** | **False Positives (n)** | **Specificity (95% CI), %** |
| --- | --- | --- | --- | --- |
| Commercial | 6 | 24 | 0 | 100 (85.8–100) |
| CCGA | 583 | 1204 | 9 | 99.3 (98.6–99.7) |

CCGA, Circulating Cell-free DNA Genome Atlas study; CI, confidence interval.
